# Integrating morphometric and climatic variables into the mapping of land use and vegetation cover in the Caatinga biome

**DOI:** 10.1007/s10661-026-15949-z

**Published:** 2026-09-29

**Authors:** Alíbia Deysi Guedes da Silva, Sara Fernandes Flor de Souza, Rebecca Luna Lucena, João Santiago Reis

**Affiliations:** 1 Graduate Programme in Geography - GEOCERES, Federal University of Rio Grande do Norte, Caicó, Brazil; 2 Graduate Programme in Geography - GEOCERES, Federal Rural University of Pernambuco, Recife, Brazil

**Keywords:** Dry forest, Random forest, Semi-arid, Classification

## Abstract

Recent landscape transformations resulting from the replacement of natural areas with anthropogenic uses have had an impact on the semi-arid region of Brazil. The objective of the research was to evaluate a land use and land cover classification model in the Caatinga biome, using machine learning techniques integrated with multiple environmental and climatic covariates. The area was segmented into six subunits, based on a combination of hydrographic regions and geomorphological domains. The random forest algorithm on the Google Earth Engine platform was used to process the supervised classification, considering predictor variables, including spectral bands and indices, tasseled cap transformation, image fractions, surface temperature, precipitation, morphometric variables, and geographic location. The results indicated that geomorphological compartmentalization increased separability between land use classes and bare soil. The model achieved an average global accuracy of 0.83 and a kappa index of 0.80. The forest vegetation and rivers, lakes, and ocean classes showed high precision, while salt marshes and herbaceous restinga indicated inconsistencies. The most relevant variables were spatial position, altitude, and climatic conditions. In this perspective, incorporating this set of elements into the machine-learning classifier proved to be an innovative and scientifically relevant approach.

**Supplementary Information:**

The online version contains supplementary material available at https://doi.org/10.1007/s10661-026-15949-z.

## Introduction

Land use and land cover reflect the interactions between social and environmental dynamics and are fundamental elements for understanding the impacts of human activities on the environment. The mosaics of anthropogenic uses and natural land covers materialized in geographic space and landscape (Bertrand, 2004). Understanding the existing patterns on the surface, aligned with the socioeconomic context, assists in the planning of natural resources, sustainable environmental management, and various scientific applications (Accioly et al., 2017).

Drylands, which cover nearly half the planet (> 40%), are characterized by water scarcity and high climate variability, and they support biodiversity, ecosystem services, and the livelihoods of millions of people (FAO, 2019; IPCC, 2021). In South America, these environments range from hyper-arid regions, such as the Atacama Desert in Chile, to semi-arid regions, such as northeastern Brazil. In this context, the Caatinga constitutes the primary vegetation type associated with the Brazilian Semi-Arid region and stands out as the country’s only unique biome. It is classified as a Seasonally Dry Tropical Forest, located in the Brazilian Semi-arid region, and presents high biodiversity, with a significant number of endemic species (Pfaltzgraff and Torres 2010). However, its natural resources have been exploited for five centuries, through the extraction of firewood, mining, grazing, and monoculture (Moreira et al., 2019; Silva & Barbosa, 2017), in addition to the advancement of urban infrastructure and controlled burning (Vasconcelos et al., 2024). In the Caatinga biome of Rio Grande do Norte (CAARN), agricultural activities predominate activities, often with inadequate management, considerably impact natural cover. Currently, new activities have stood out in relation to initiatives to diversify the energy matrix, such as photovoltaic and wind energy projects (Dantas et al., 2019). Considering forecasts related to climate change and the impacts of human actions on the region (IPCC, 2021), it is necessary to expand research focused on the analysis of spatial patterns of socioenvironmental organization as a basis for decision-making.

In an interactive process, the type and spatial distribution of vegetation respond to the climatic, edaphic, and topographic characteristics of each geoenvironment (He, 2014; Oliveira et al., 2019). The physical environment of the CAARN presents diverse landforms, such as plateaus, plains, depressions, and tablelands, over sedimentary strata of the Potiguar Basin and ancient terrains of the crystalline basement, which are found at varying altitudes (Pfaltzgraff and Torres 2010). These geological regions therefore exhibit distinct geomorphological features, soil properties, and terrain conditions (Oliveira & Medeiros, 2018). These contrasts occur along a marked climatic gradient, ranging from semi-arid conditions across most of the state to dry and humid sub-humid environments toward the eastern coast, with significant spatial and temporal variability in precipitation (Lucena et al., 2018; Silva et al., 2022a, b). Consequently, differences in soil depth, texture, drainage, fertility, and water-holding capacity, along with variations in topography and water availability, contribute to the spatial differentiation of vegetation types and land use and land cover (LULC) patterns.

From this perspective, incorporating a set of environmental elements, organized into geomorphological units, can enhance the reconstruction of real space and the discrimination of terrestrial targets, helping to resolve spectral ambiguities. Studies have demonstrated the potential of integrating environmental variables, such as precipitation and temperature, into the classification of surface targets in different regions of Brazil (Silva et al., 2024a, b; Zeferino et al., 2020); however, this approach is still in its infancy in the context of CAARN. However, scientific studies on land use and land cover classification still rely predominantly on spectral data and derived indices (Franca Rocha et al., 2024; Jin et al., 2018; Macarringue et al., 2023; Souza et al., 2020). Accordingly, implementing environment and climate representative layers within the machine-learning classifier constitutes an innovative and scientifically relevant approach.

The introduction of artificial intelligence algorithms based on machine learning has optimized the identification and extraction of information from targets on the Earth’s surface. Tree-based algorithms, such as random forest (RF), learn to make decisions based on an initial condition (Breiman, 2001). This architecture is widely used in supervised classification as a technique for predicting distinct spectral characteristics, such as land use and land cover (Fentaw & Abegaz, 2024; Macarringue et al., 2023; Marzulo Maia Martins et al., 2024).

Currently, the largest initiative of this kind is the Annual Land Cover and Land Use Mapping Project in Brazil (MapBiomas), which was launched in 2015 and relies on a collaborative network to generate multitemporal classification maps dating back to 1985. However, the overall accuracy at legend level 2 is 79.4% (collection 10) for the Caatinga biome. This result highlights that there are still challenges in the search for more effective methodologies for the automatic classification of large, heterogeneous areas, especially with regard to improving accuracy.

Given this scenario, this study sought to investigate two central questions: (i) to what extent is a model generated by a machine learning algorithm effective in the digital classification of extensive areas with heterogeneous spatial patterns, such as the Caatinga biome in the state of Rio Grande do Norte? and (ii) how do environmental variables influence the spatial behavior of this vegetation? It is assumed that segmenting the area into sedimentary and crystalline geoenvironments contributes to reducing spectral confusion in the classification process. Furthermore, it is presumed that environmental variables, such as rainfall, surface temperature, and morphometric parameters, exert a significant influence on the spatial behavior of the vegetation of the Caatinga biome.

Thus, the objective was to evaluate a land use and land cover classification model in the Caatinga biome, in the state of Rio Grande do Norte, for the year 2023, using machine learning techniques integrated with multiple environmental and climatic covariates.

## Materials and methods

### Study area

**Fig. 1:**
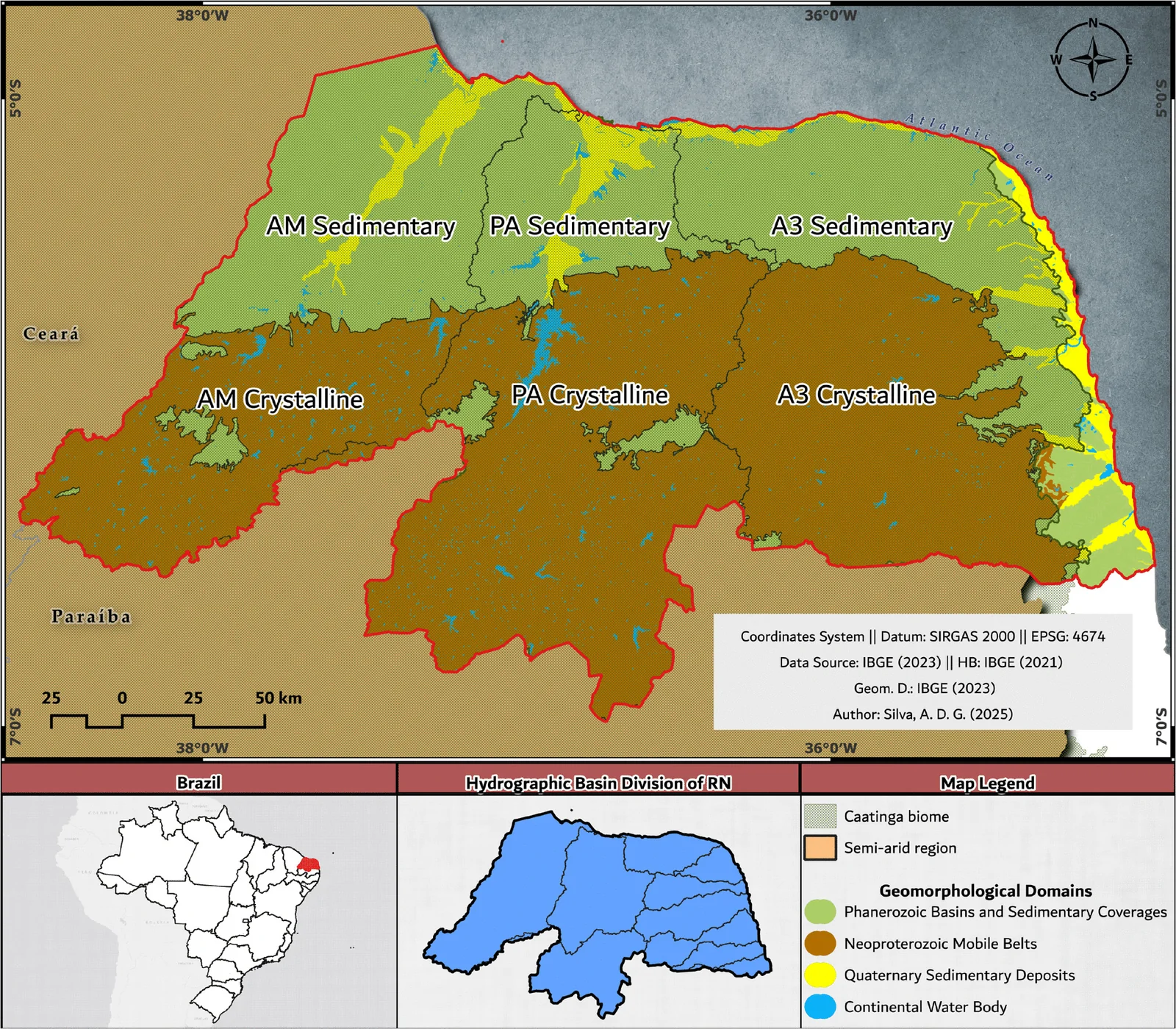
Map of the state of Rio Grande do Norte highlighting the divisions of the study area

The state of Rio Grande do Norte is a federative unit of Brazil located in the northern corner of the Northeast region, bordering the states of Ceará to the west, Paraíba to the south, and the Atlantic Ocean to the north and east of its territory. Regarding the climate, it can be divided into three main climatic domains: semi-arid, dry sub-humid, and humid (Fig. 1).

The semi-arid domain encompasses 155 of the 167 municipalities and is particularly pronounced in the central portion of the state and along parts of the northern coast. It is characterized by low and irregular rainfall, high interannual variability, elevated atmospheric evaporative demand, and a persistent water deficit. Rainfall over the northern and inland sectors is primarily controlled by the seasonal displacement of the Intertropical Convergence Zone, whereas the eastern coast is also influenced by Atlantic easterly disturbances and trade-wind moisture transport. Dry sub-humid conditions occur locally in western highlands and in the Agreste, a transitional zone between the semi-arid interior and the humid eastern coast, where rainfall is more frequent and better distributed (Lucena et al., 2018; Silva et al., 2022a, b).

The Caatinga biome covers approximately 50,746.485 km^2^ and predominates throughout the state. Hyperxerophilous Caatinga, with a relatively open and strongly drought-adapted structure, occupies the driest areas, whereas denser hypoxerophilous physiognomies occur mainly at higher elevations and in the Agreste. The eastern coast contains Atlantic Forest and transitional formations, while restinga vegetation, dune fields, and mangroves occur along the coast and in estuarine environments (de Araújo Filho et al., 2022).

The principal geomorphological domains are the Phanerozoic Basins and Sedimentary Covers, the Neoproterozoic Mobile Belts, and the Quaternary Sedimentary Deposits (IBGE, 2023). They encompass coastal and fluvial plains, tablelands, plateaus, gently dissected erosional surfaces, residual uplands, and inselbergs. The sedimentary sectors generally have flatter and less rugged terrain and deeper soils, whereas the crystalline sector consists largely of an ancient, slightly dissected erosional surface interrupted by inselbergs and residual massifs. Elevated areas exceeding 700 m, such as the Serra de Santana and Serra de Martins, generate locally cooler and wetter conditions.

Climate, parent material, and relief jointly control soil development. Across most of the semi-arid interior, the water deficit restricts leaching and chemical weathering, favoring base-rich soils with weak to moderate pedogenic development. Deeper and more strongly weathered soils occur on old and stable geomorphic surfaces and in more humid environments. Geological diversity, and the resulting pedodiversity expressed across the state’s morphosculptural domains, therefore influence the LULC mosaic by controlling soil depth, texture, fertility, drainage, water storage, and terrain accessibility. Consequently, extensive livestock production and rainfed agriculture are widespread in the semi-arid interior, whereas flatter sedimentary and alluvial areas support more intensive or irrigated agriculture. Salt production and aquaculture occur in saline estuaries, while agriculture, urban expansion, infrastructure, and tourism exert greater pressure on the humid eastern coast.

To represent these environmental contrasts, the study area was divided by intersecting geomorphological domains with hydrographic-basin subdivisions. The Apodi-Mossoró basin was separated into AM Crystalline and AM Sedimentary, and the Lower and Middle Piranhas–Açu basin into PA Crystalline and PA Sedimentary. The Camurupim/Cabelo, Maxaranguape, Ceará-Mirim, Potengi, Trairi, Jacu, and Curimataú basins were grouped into a third area, subsequently divided into A3 Crystalline and A3 Sedimentary (Fig. 1). Sedimentary terrains of different geological ages were merged at the analytical scale because they share broad morphostructural characteristics, although their geological histories and landscape evolution differ.

The sedimentary sector extends across the northern and eastern portions of the state and corresponds mainly to the Phanerozoic Basins and Sedimentary Covers domain, with Quaternary Sedimentary Deposits along the coast, estuaries, and recent fluvial and marine plains (IBGE, 2023). The AM Sedimentary unit occupies its western portion and contains predominantly Ferralsols, Cambisols, Chernozems, and Phaeozems, according to the World Reference Base for Soil Resources (IUSS Working Group WRB, 2022; Jacomine et al., 1971). Ferralsols and Cambisols occur mainly on Mesozoic and Cenozoic covers, whereas Chernozems and Phaeozems are closely associated with the Upper Cretaceous carbonate rocks of the Jandaíra Formation (Bertotti et al., 2017). In the Quaternary deposits of the Apodi-Mossoró estuary, poorly drained Gleysols may contain high concentrations of soluble salts, consistent with the hypersaline conditions produced by high evaporation and limited freshwater discharge (Jacomine et al., 1971; Medeiros et al., 2018).

The PA Sedimentary unit, in the central portion of the Phanerozoic Basins and Sedimentary Covers domain, is dominated by Cambisols, Arenosols, Acrisols, and Lixisols developed from Mesozoic and Cenozoic sedimentary materials (Jacomine et al., 1971). Their distribution reflects differences in sediment texture, geomorphic-surface age, drainage, and weathering intensity. Gleysols with high salt contents occur locally within the Quaternary deposits of the Piranhas-Açu estuary. These edaphic contrasts contribute to a mosaic of Caatinga vegetation, grazing land, rainfed agriculture, and irrigated cropping, whereas the hypersaline fluvial-marine plains of the lower Açu River are extensively occupied by salt ponds (Diniz and Vasconcelos 2017).

The A3 Sedimentary unit encompasses the eastern part of the Phanerozoic Basins and Sedimentary Covers domain and a coastal strip of Quaternary Sedimentary Deposits (IBGE, 2023). It differs from the other sedimentary units because of its more humid climate and transitional position between the Caatinga and Atlantic Forest biomes. Ferralsols predominate on stable Cenozoic surfaces of the coastal tablelands, whereas Arenosols occur on coastal plains and dune systems (Jacomine et al., 1971). Variations in rainfall and drainage along the Barreiras Formation also result in contrasting pedogenic pathways, including ferrallitization (Rêgo et al., 2019). Greater rainfall, deeper soils, and low terrain ruggedness favor denser vegetation and more intensive land occupation, resulting in a fragmented landscape composed of forest remnants, restinga vegetation, mangroves, agricultural areas, and urban infrastructure.

The crystalline sector corresponds predominantly to the Neoproterozoic Mobile Belts geomorphological domain. However, this broad designation encompasses a complex geological mosaic of Archean, Paleoproterozoic, and Neoproterozoic crustal components and therefore does not imply geological uniformity (Oliveira & Medeiros, 2018). Compared with the sedimentary sector, its greater terrain variability and larger proportion of shallow and rocky soils strengthen the influence of parent material and relief on vegetation and land use. Leptosols and shallow Regosols are particularly important in the Brazilian semi-arid region because their limited effective depth and water-storage capacity increase environmental constraints and susceptibility to erosion (Pinheiro Junior et al., 2022).

The AM Crystalline unit is underlain mainly by Paleoproterozoic rocks, with a smaller contribution from Neoproterozoic units, and is dominated by Lixisols, Luvisols, and Regosols (Jacomine et al., 1971; Oliveira & Medeiros, 2018). Small residual tablelands, including the Serra de Martins and Serra de Portalegre, form local sedimentary enclaves within the predominantly crystalline landscape. These elevated surfaces are capped by Cenozoic sediments of the Serra dos Martins Formation and contrast with the less-developed soils of the surrounding crystalline lowlands (Souza et al., 2025). This variation in elevation, parent material, and soil properties produces a heterogeneous landscape comprising open Caatinga, denser upland vegetation, grazing land, and agricultural areas (Menezes et al., 2021).

The PA Crystalline unit also belongs predominantly to the Neoproterozoic Mobile Belts domain and comprises a heterogeneous assemblage of Paleoproterozoic and Neoproterozoic rocks within the Rio Piranhas-Seridó tectonostratigraphic domain (Medeiros et al., 2021). Luvisols, Leptosols, and Regosols form a complex soil association, particularly on shallow weathering mantles, rocky terrain, and inselbergs (Jacomine et al., 1971). Ferralsols occur locally on the residual tablelands of Serra de João do Vale and Serra de Santana, where Cenozoic covers of the Serra dos Martins Formation overlie the crystalline basement (Morais Neto et al., 2008). Studies in Serra de Santana have confirmed the occurrence of deeper and more strongly developed soils under milder upland edaphoclimatic conditions (Farias et al., 2018). In the surrounding lowlands, shallow soils and restricted water availability limit intensive agriculture and favor Caatinga vegetation interspersed with extensive livestock production and rainfed agriculture.

The A3 Crystalline unit is included in the Neoproterozoic Mobile Belts domain but contains a heterogeneous substrate composed of Neoproterozoic, Paleoproterozoic, and Archean rocks (Oliveira & Medeiros, 2018). Its proximity to the eastern coast also results in comparatively more humid conditions. Planosols predominate, followed by Leptosols, Luvisols, and Lixisols (Jacomine et al., 1971). The formation and properties of Planosols and Luvisols in the Borborema Province are strongly controlled by the weathering of their metamorphic parent rocks, while rainfall gradients regulate mineral alteration and Planosol development (Câmara et al., 2021; Sousa et al., 2023). Restricted drainage in Planosols, shallow profiles in Leptosols, and variations in rainfall and parent material contribute to a mosaic of hypoxerophilous Caatinga, transitional Atlantic Forest vegetation, pastures, rainfed agriculture, and urbanized areas.

Thus, the six units represent integrated environmental strata rather than exclusively geological or hydrographic subdivisions. The contrast between sedimentary and crystalline terrains controls soil depth, texture, mineralogy, drainage, and terrain conditions, whereas the west-east climatic gradient regulates water availability, weathering intensity, and vegetation density (Câmara et al., 2021; Pinheiro Junior et al., 2022; Rêgo et al., 2019). These environmental controls interact with the conversion of Caatinga into pastures and croplands, providing the basis for the spatial differentiation of LULC across Rio Grande do Norte (Menezes et al., 2021).

### Data acquisition and methods used

**Fig. 2:**
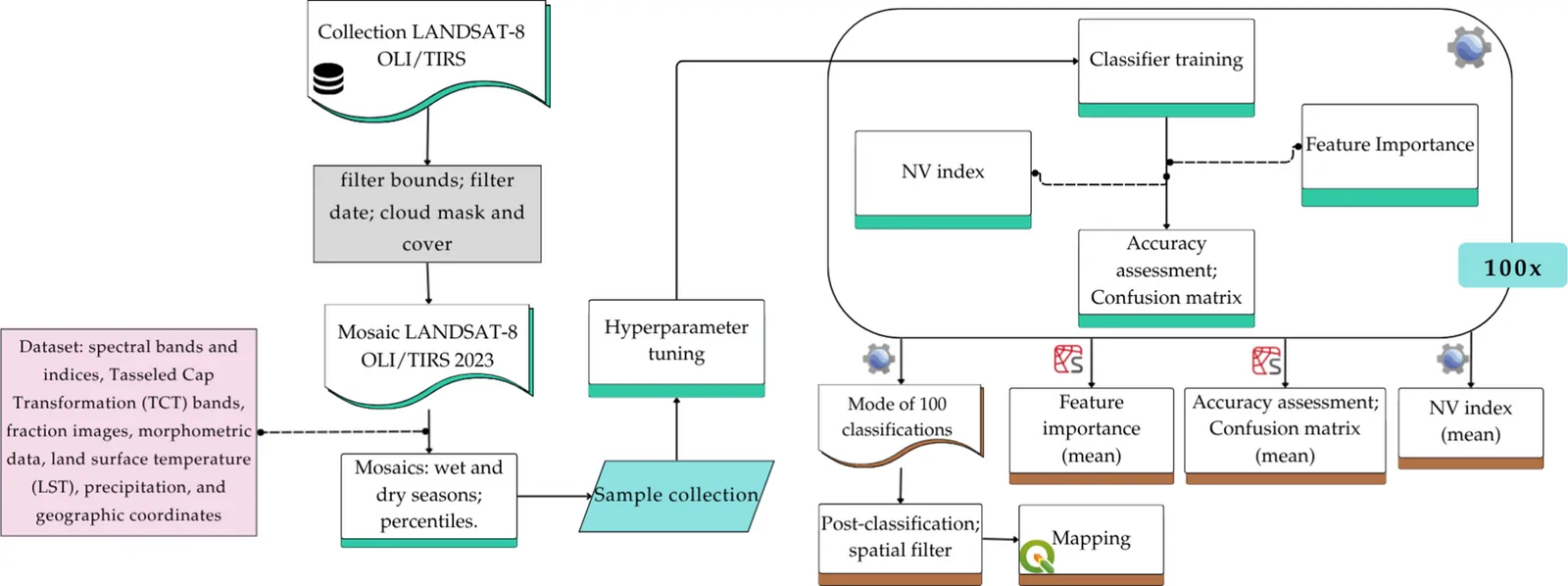
Methodological scheme for land use and land cover classification

To understand the spatial distribution patterns of natural and artificial landforms present in the CAARN, the main methodological procedures are presented in steps in the flowchart (Fig. 2). In terms of materials, the collection of images referring to the top of the atmosphere (TOA) from the Landsat-8 OLI sensor was used, which were converted into surface reflectance (SR). The execution of the predominant steps took place directly in the Google Earth Engine (GEE), a cloud-based platform designed with high capacity for acquiring and processing spatial data (Gorelick et al., 2017).

The procedures were applied individually to each fragment of the study area. Satellite images underwent area filtering, cloud cover percentage (50%), application of algorithms for cloud and shadow removal/reduction, and temporal filtering (2023). Data analysis, statistical calculations, and creation of visual graphs were performed in the open-source IDE Spyder. For this, the use of some libraries was necessary: Matplotlib (Caswell et al., 2020), pandas (McKinney, 2010), geoPandas (Jordahl et al., 2020), numpy (Harris et al., 2020), and sea-born (Waskom, 2021). Vector data processing and final map creation were performed in the QGIS software (version 3.34-Prizren).

### Sample data and land use and land cover classes

Image classification involved the process of collecting samples grouped into classes manually using point geometry in GEE. The number of samples varied according to the representativeness of the different classes in each area. In addition to the manual points, 59 points collected in the field and 271 points made available by MapBiomas (Souza et al., 2020) for the year 2023 and adapted for this research were included.

The collection of field coordinates was conducted using a Garmin Montana 650 GPS receiver, compass, and visual records of the landscape. For this stage, the selective non-random sampling technique was adopted, in which the sampling points are defined arbitrarily, based on the preliminary classifications. In addition to direct observation and photographic recording, mobile applications such as Avenza, Google Maps, and GPSMapCamera supported navigation and documentation of the observed classes. This information was fundamental for the reinterpretation of the classifications and allowed for the readjustment of the mappings.

**Fig. 3:**
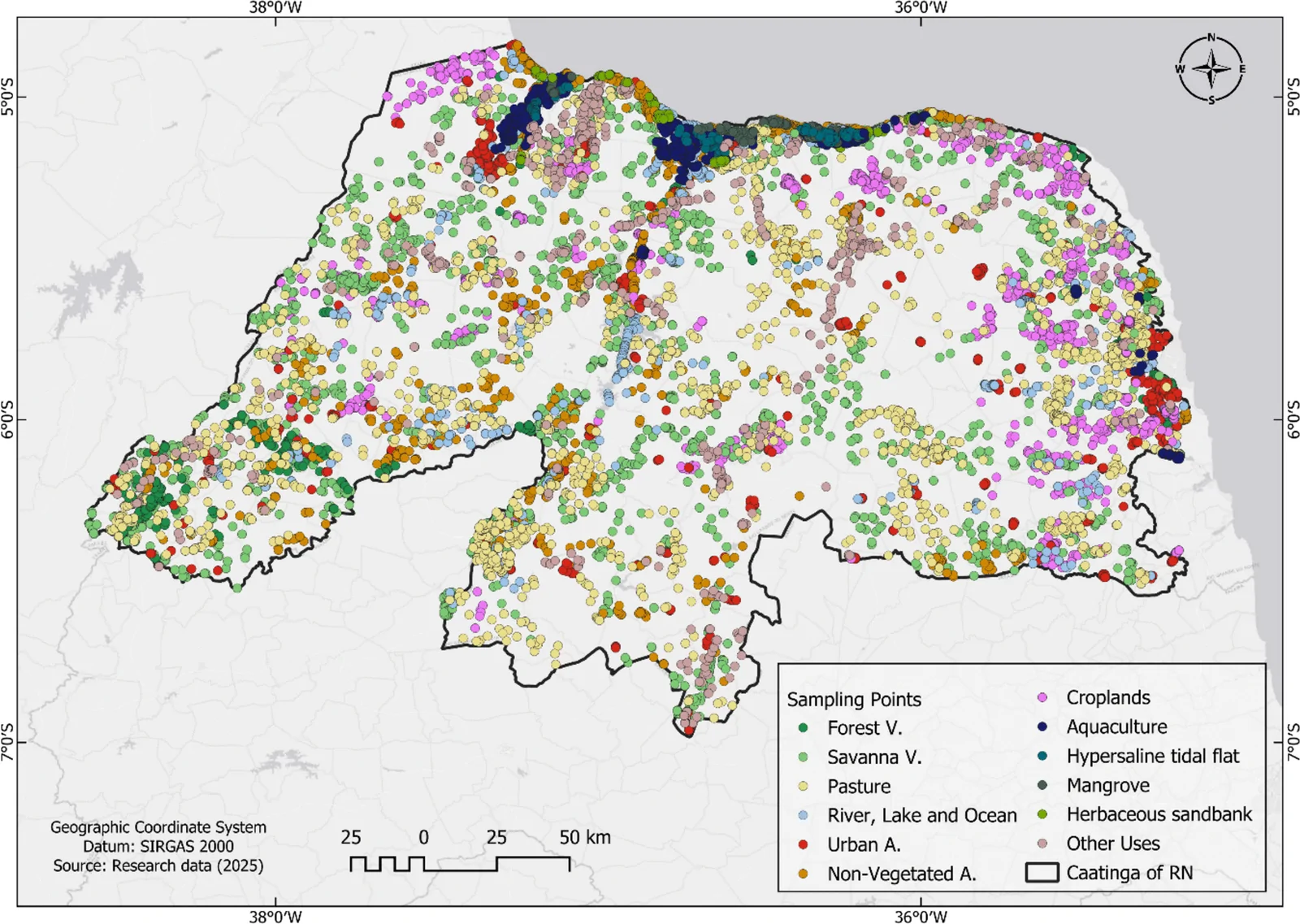
Spatial distribution of land use and cover samples

The sampling methods used in this research were visual interpretations of Landsat scenes and field surveys. Samples were manually extracted using point geometry on the GEE platform, based on the false-color RGB mosaic (R6G5B4). To provide support during photointerpretation, land use and land cover mapping materials from IBGE and MapBiomas collection 9 were selected (Souza et al., 2020). Field observations were used to aid in the interpretation and verification of the targets. A total of 11,079 samples were collected, covering the different classes and the analyzed area (Fig. 3). The number and location of the sampling points were determined based on the identification and spatial representation of each class.

The theoretical and methodological basis for defining the analysis nomenclatures was the IBGE Land Use Classification System (LUCS) (IBGE, 2012) and the MapBiomas hierarchical classification (Souza et al., 2020). Thus, with a view to discerning the main vegetation physiognomies and different types of uses and covers, the classes were framed into five categories at level I and thirteen at level II: (1) Non-Agricultural Anthropic Areas, which includes (i) Urban Areas and (ii) Aquaculture; (2) Agricultural Anthropic Areas, composed of (iii) Croplands and (iv) Pasture; (3) Natural Vegetation Areas correspond to (v) Forest Vegetation, (vi) Savanna Vegetation, (vii) Mangrove, (viii) Herbaceous Restinga (Sandbank V.), and (ix) Apicum (hypersaline tidal flat); (4) Water Bodies include (x) Rivers, lakes/lagoons, and ocean; and (5) Other Areas, referring to (xi) Non-vegetated areas, (xii) Other Uses, and (xiii) Not Observed. The classification key and the number of samples per class are given in Table S1.

### Set of predictor variables

The mapping of land use and vegetation cover for the year 2023 was performed using the RF algorithm. This period corresponds to the most recent year for which complete data is available. It supports a set of distinct variables as input parameters. Therefore, the research applied several spectral indices related to vegetation cover, leaf area, chlorophyll, and biomass Caatinga environment. Thus, the input parameters for classification correspond to spectral bands (B2, B3, B4, B5, B6, and B7), spectral indices, fraction images derived from the Linear Spectral Mixture Model (LSMM), tasseled cap transformation (brightness, greenness, and wetness), geomorphological variables, climatic variables, and spatial position.

Due to the cyclical influence of precipitation on the physiology of the Caatinga and the spatial variability of rainfall, climatological normals (from Brazilian National Institute of Meteorology—INMET) were analyzed for the three major research areas, dividing them into two periods corresponding to the months of increasing rainfall volume and those in which there is a decline in monthly totals. Thus, for the Apodi-Mossoró and Piranhas-Açu basins, the rainy season ran from January to June, while for the basin composition between the agreste and eastern mesoregions, this period was from January to August. The dry season lasted until December. Furthermore, the variables underwent statistical percentile reduction (25, 50, 75, and 90), resulting in 153 bands used to train the machine learning models. Given the model’s high performance on correlated data (Breiman, 2001), the entire dataset was used (Table 1).

**Table 1:** Predictor variables used in the model

Name (variable)	Description	Formula	Reference
Normalized difference vegetation index (NDVI)	Estimation of the quantity and vigor of vegetation	(NIR − RED)/(NIR + RED)	Rouse et al., 1974
Normalized difference water index (NDWI)	Water content estimate	(GREEN − NIR)/(GREEN + NIR)	Gao, 1996
Soil-adjusted vegetation index (SAVI)	Vegetation index adjusted to minimize the influence of soil in areas with little vegetation cover	(NIR − RED)/(NIR + Red + L) × (1 + L); L = 0.5	Huete, 1988
Modified soil-adjusted vegetation index (mSAVI)	Modified version of SAVI to increase accuracy in exposed soils	[(2 × NIR + 1) − √(2 × NIR + 1) 2 − 8 × (NIR − RED)]/2	Qi et al., 1994
Green soil-adjusted vegetation index (gSAVI)	SAVI version that uses the green band to highlight vegetation	(NIR − GREEN)/((NIR + GREEN + 0.5) × (1 + 0.5))	Sripada et al., 2006
Normalized Difference Built-up Index (NDBI)	Index for identifying urban and built-up areas	(SWIR − NIR)/(SWIR + NIR)	Zha et al., 2003
Green Normalized Difference Vegetation Index	NDVI version that uses the green band to assess vegetation	(NIR − GREEN)/(NIR + GREEN)	Gitelson et al., 1996
Enhanced vegetation index (EVI)	Estimation of densely vegetated areas	2.5 × (NIR − RED)/(NIR + 6 × RED − 7.5 × BLUE + 1)	Liu & Huete, 1995
Difference vegetation index (DVI)	Simple vegetation index	NIR − RED	Tucker, 1979
Modified normalized difference water index (mNDWI)	A variant of NDWI, adjusted to identify water bodies	(GREEN − SWIR)/(GREEN + SWIR)	Xu, 2006
Moisture stress index (MSI)	It measures water stress in vegetation	SWIR1/NIR	Hunt Jr & Rock 1989
Chlorophyll Vegetation Index (CVI)	Evaluates chlorophyll concentration	(NIR/GREEN) × (RED/GREEN)	Vincini et al., 2008
Green Ratio Vegetation Index (GRVI)	Index based on the proportion of green to assess vegetation	(NIR/GREEN)	Motohka et al., 2010
Modified Simple Ratio (MSR)	A modified version of the Simple Ratio for assessing vegetation health	((NIR/RED) − 1)/(sqrt(NIR/RED) + 1)	Chen, 1996
Ratio Vegetation Index (RVI)	Estimation of the proportion of vegetation	(NIR/RED)	Richardson & Wiegand, 1977
Normalized Burn Ratio (NBR)	Index used to detect burned areas	(NIR-SWIR)/(NIR + SWIR)	Key & Benson, 2006
Normalized Difference Tillage Index (NDTI)	Index for detecting soil management practices, such as cultivation	(SWIR1 − SWIR2)/(SWIR1 + SWIR2)	Van Deventer et al., 1997
Modified Photochemical Reflectance Index (MPRI)	Vegetation estimate	(GREEN − RED)/(GREEN + RED)	Yang et al., 2008
MDE (slope, hillshade, elevation, aspect)		-	SRTM
Spectral Linear Mixture Model (fraction_solo, fraction_agua, fraction_vege)	Soil, water, and vegetation ratio	-	Shimabukuro & Smith, 1991
Tasselled cap (vegetation, wetness, brightness)		-	Kauth & Thomas, 1976
Spectral bands (B2, B3, B4, B5, B6)	Spectral bands of different wavelengths	-	Landsat-8
Precipitation (precipitacao_2023)	Estimated precipitation data for 2023	-	Silva et al., 2024a
Land surface temperature (LST_2023)	Earth’s surface temperature in 2023	-	Jesus & Santana, 2017
Geographical position (latitude, longitude)	Measurements of position on the Earth’s surface	-	-

### Estimated precipitation and land surface temperature (LST)

The estimated precipitation layer, used as a predictor in land use and land cover (LULC) classification, as developed by Silva et al. (2024a), was derived from a RF regression model based on 2023 annual precipitation observations from the Empresa de Pesquisa Agropecuária do Rio Grande do Norte (EMPARN). The original dataset comprised precipitation records from telepluviometers and automatic rain gauges (198 stations with complete annual records). Precipitation observations were modeled as the dependent variable, using environmental covariates: elevation, slope, aspect, and hillshade, derived from the SRTM MDE (30 m); NDVI and SAVI, derived from Landsat-8 OLI images (30 m); LST, derived from Landsat-8 thermal data; as well as latitude, longitude, relative distance variables, air temperature, relative humidity, and the De Martonne aridity index.

For precipitation modeling, the covariates were combined into a single multiband dataset, and their values were extracted from the 198 rain gauge stations. The observations were randomly split into 80% for training and 20% for validation, and the RF model was configured for regression, with 500 trees and no limit on the maximum number of nodes. The procedure was repeated 100 times, using different random seeds to reduce the influence of the random partitioning of the observations, and the final precipitation map was obtained by averaging the forecasts from the 100 runs.

LST was calculated according to the equation described in Jesus and Santana (2017), which uses the Landsat thermal band and NDVI index.

### Classification stage

The random forest model (Breiman, 2001) is characterized as a nonparametric classifier in the supervised machine learning category. It consists of an ensemble method that combines a set of K decision trees (a forest). The main technique is bagging (bootstrap aggregating), in which, for 100 trees, 100 subsets are created by random sampling with replacement from the original dataset. In addition, each tree is trained using a different set of variables (Breiman, 2001). The samples not selected for training are called “out-of-bag” (OOB), and their composition can be configured (Chang & Bai, 2018). The bagging method selects both the data randomly (bootstrap sampling) and the variables that will compose a given decision tree. This randomness is important to prevent the model from suffering from overfitting, in other words, to prevent the model from learning the existing data too perfectly and failing to predict new outcomes (Hastie Robert & Friedman, 2009).

In this study, RF was implemented in GEE to classify land use and land cover in CAARN, using spectral, climatic, topographic, and location predictors. Hyperparameter tuning in machine learning models is crucial for determining model performance (Géron, 2019). The calibration of the number of trees (ntree) was performed using the grid search method, simultaneously considering the out-of-bag (OOB) error inherent to RF and the overall accuracy. Schematically, the code tested ntree values ranging from 100 to 2000. This experiment was performed 10 times for all studied area units, and the best result for average overall accuracy was selected. ntree controls the size of the ensemble (Breiman, 2001).

The remaining hyperparameters were set to the default values of the “smileRandomForest” implementation. Thus, “mtry” was set to the square root of the total number of variables (12.3), “nodesize” was set to 1, and “bag fraction” was set to 0.5.

Due to the inherent randomness of the algorithm in selecting training and validation samples, the script was programmed to execute all classification steps in 100 repetitions (De Castro Paes et al., 2023). Thus, the model was trained and evaluated several times using a different seed in each iteration. This procedure resulted in 100 maps for each fragment of the study area. The consolidation of the rounds was performed using the mode, generating a final classification based on the class most frequently assigned by the trees in the prediction process.

The feature importance (FI) resource extracted from the RF algorithm was used to evaluate the relevance of each variable and its contribution to class distinction. FI is an internal metric that estimates, during training, how much each layer influences the overall performance of the model. This measure is based on the reduction of impurity in a node of the decision tree when the variable is used, known as mean decrease in impurity (MDI) or Gini impurity index (Breiman, 2001).

### Post-classification procedures

#### Evaluation of model performance and classification uncertainty measure

Statistical accuracy assessment is a fundamental component in map production using classifiers. In this research, each division of the area was evaluated using test samples to assess the accuracy of the machine learning algorithm. The confusion matrix or contingency table is the first step, which consists of comparing the classification obtained with the external validation set, and allows evaluating the classification performance. Randomly, 70% were allocated to training and 30% to external validation of the classifier (Congalton & Green, 2019). In addition, the overall accuracy, user accuracy (UA), producer accuracy (PA), and the kappa index were extracted, which is a coefficient to test the agreement between the observed results and the predicted results (Cohen, 1960).

For the uncertainty study of the 100 iterations performed, the normalized variety (NV) metric, proposed by de Araújo (2021), was adopted. NV measures the variation of classes assigned to each pixel across several iterations and normalizes this value based on the total number of possible classes as a percentage. Thus, the uncertainty is 0 when the class is the same in all 100 iterations, and the uncertainty is high when there is a greater presence of other types of classes different from the final one, i.e., the mode of the classifications. It is, therefore, a spatial indicator of the classification confidence and allows identifying areas where the model uncertainty was higher (Eq. 1), where variety is the value of the number of unique classes in each pixel across the 100 rounds, and CM is the number of grouped classes.

1 $$Normalized\;Variety\;=\frac{\left(variety-1\right)}{\left(CM-1\right)}\times100$$

#### Spatial filter

One of the limitations of pixel-based supervised digital classification is the propensity to obtain pixel noise, which can compromise the quality of the information. In the post-classification stage, the data were refined to reduce the effects of isolated pixels, using the major spatial filter (Ghimire et al., 2010). The mode was applied to smooth out spatial inconsistencies in each pixel in a 3 × 3 neighbor window.

## Results

### Influence of the adjustment on the number of trees

**Fig. 4:**
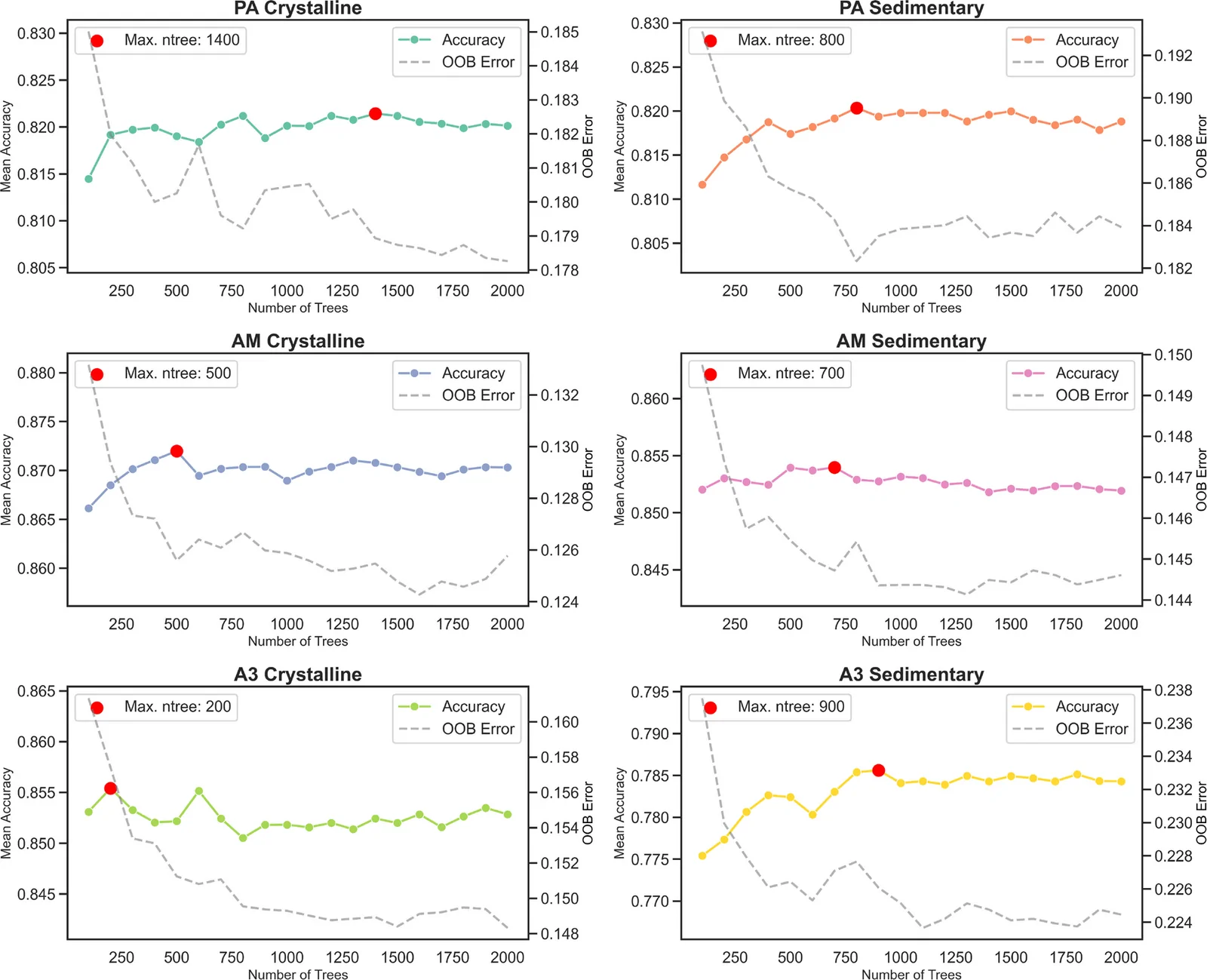
OOB and ntree results for the six sub-areas

Figure 4 presents the tests performed to find the best fit in the number of trees (ntree) and the internal errors of the classifications. The graphs demonstrate RF performance for the fragmentations of the study area (PA Crystalline, PA Sedimentary, AM Crystalline, AM Sedimentary, A3 Crystalline, and A3 Sedimentary). The metric adopted to evaluate the model was the overall accuracy as a function of the ntree. It can be observed that each scenario presents a specific ntree that maximizes the accuracy result, indicated by the red dot.

In general, the graph shows that the OOB (out-of-bag) error decreases proportionally to the increase in ntree (number of trees); however, upon reaching approximately 800 trees, the error tends to stabilize. The only exception was the PA Cristalino area, where the highest precision was obtained at ntree = 1400. Furthermore, low ntree values are associated with lower accuracies and high levels of model imprecision, except in the A3 Cristalina area, where 200 trees already provided satisfactory performance. Thus, considering both accuracy and OOB reduction, the second-best result, corresponding to 600 trees, was adopted.

### Analysis of classification uncertainties

**Fig. 5:**
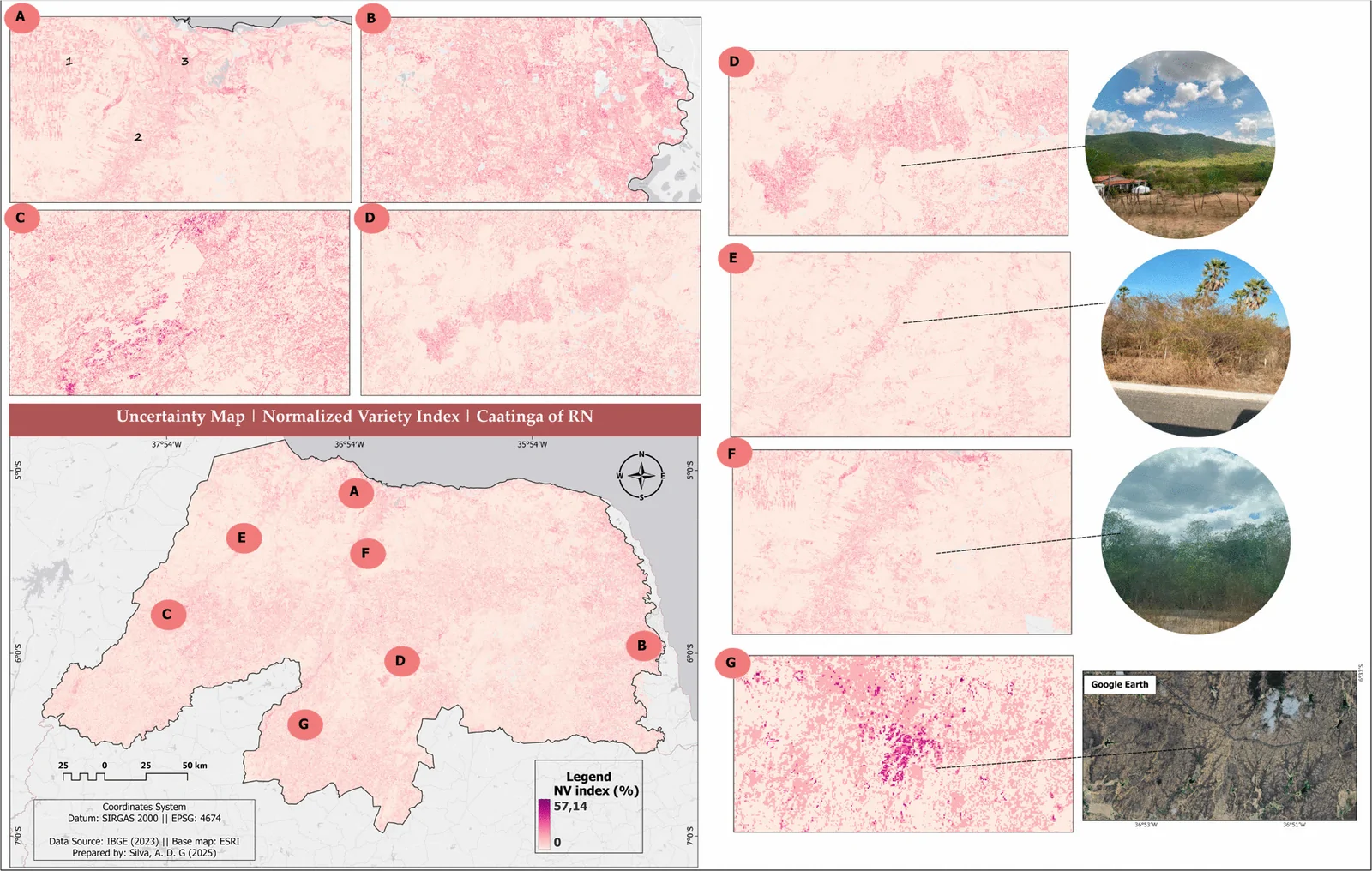
Uncertainty maps calculated using the NV index. Low NV values in areas of non-open vegetation: **A** 1. croplands in Serra do Mel, 2. irrigated fruit farming in the Piranhas-Açu basin, 3. Piranhas-Açu estuary; **B** urban complex; **C** sand near a reservoir; **D** areas of natural cover on mountain slopes and massifs; **E, F** arboreal-shrubby steppe vegetation; **G** pixels with greater uncertainties

To understand the degree of confidence in the predictions made by RF from different sample groups, an uncertainty index was applied. In general, high NV values, greater than 35%, were identified in croplands, such as Serra do Mel (Fig. 5A.1), in the irrigated fruit-growing area of the Piranhas-Açu basin (Fig. 5A.2), and in cashew and cassava production in Serra de Santana (Fig. 5D), characterized by intensive management throughout the year. Also, in the region (Fig. 4A.3), high variability in predictions was observed in the Piranhas-Açu estuarine complex, seen in the apicum and herbaceous restinga classes.

Figure 5B highlights the heterogeneity of uncertainties in areas of high and medium urban planning, such as Parnamirim, São José de Mipibu, and Macaíba (São Paulo, 2016). The confusion table indicated that 36 samples of urban areas were incorrectly classified as Other Anthropic Uses. This occurs due to the spectral diversity of different cover types present in the same pixel. Figure 5C shows an example of uncertainties in sandy areas adjacent to the Santa Cruz do Apodi reservoir, where the sandy soil, lacking vegetation cover, was sometimes mistaken for areas of anthropogenic or urban use, as also recorded in the error matrix.

In contrast, in areas of natural cover, such as the slopes of mountain ranges and massifs (Fig. 5D.1), where the vegetation is generally dense, the model recorded low variations. In the mid-western portion of the Apodi-Mossoró basin (Fig. 5E), arboreal-shrubby steppe vegetation was identified, with a low to medium degree of stratification openness, a pattern similar to that recorded in the municipality of Upanema. Similar characteristics were recorded in the vegetation of the municipality of Alto do Rodrigues, in the PA basin (Fig. 5F). Both areas are in sedimentary formations with flat relief.

It is important to highlight that the greatest uncertainties were recorded in areas of crystalline basement. An example was identified in the municipality of Jardim do Seridó, in an area of vegetation cover classified as park savanna-steppe and grassy-woody savanna-steppe (Fig. 5G), corresponding to the pasture class, located in the Seridó microregion. The eastern sector of this microregion is recognized as one of the desertification nuclei in Brazil, while the rest of the area shows high susceptibility to this process.

### Accuracy analysis of classifications by geomorphological compartmentalization

**Fig. 6:**
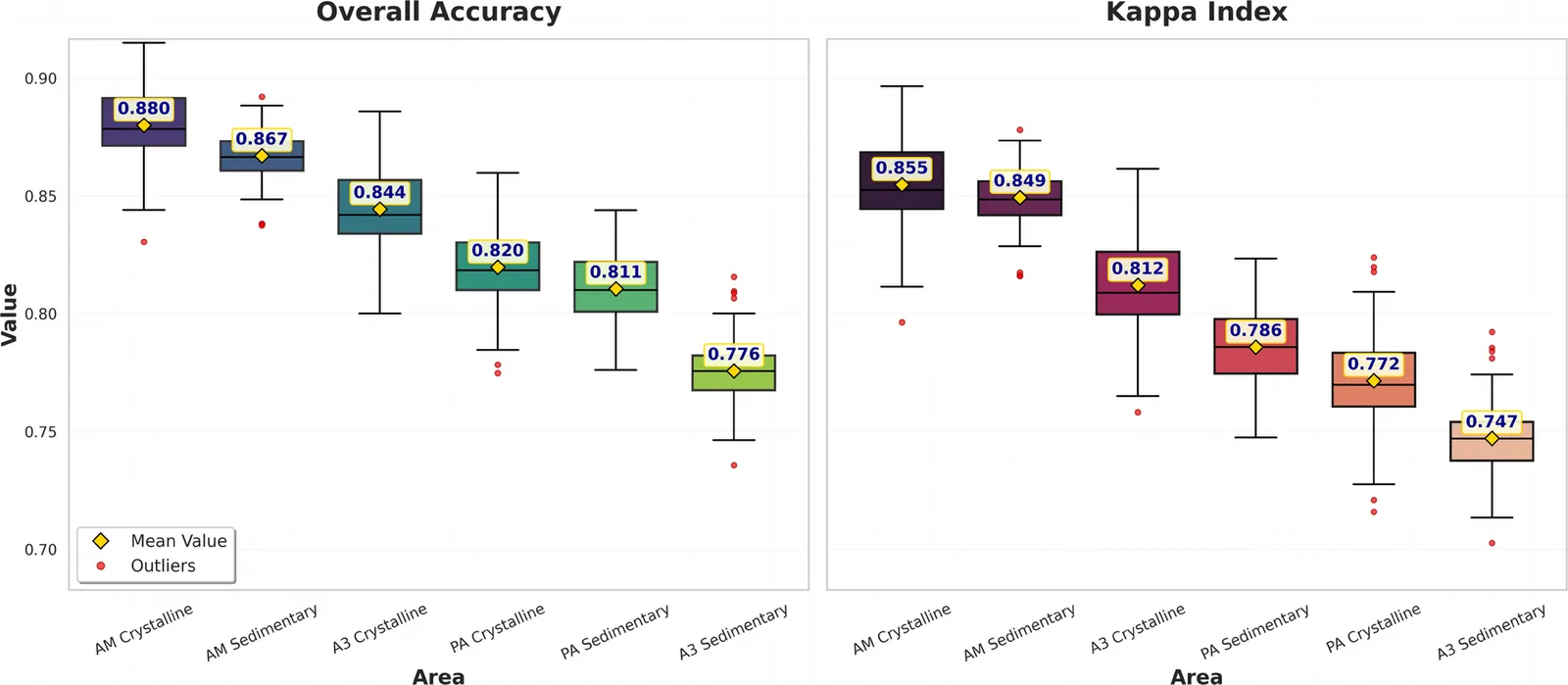
Results of overall accuracy (**a**) and kappa index (**b**) by geomorphological compartments

Based on the performance metrics applied to each segmented area, the results indicate that the methodology employed produced distinct yet satisfactory responses. Figure 6 presents the boxplots corresponding to the kappa index and overall accuracy (OA) results, whose values consider the 100 iterations performed by the algorithm.

The highlighted values correspond to the averages obtained, with accuracy metrics ranging from 78 to 88% and a kappa index between 0.75 and 0.85. The predictions for the AM Crystalline, AM Sedimentary, and A3 Crystalline areas were classified as excellent, according to the kappa index. The sub-areas of the Piranhas-Açu basin (PA Sedimentary and PA Crystalline) and the A3 Sedimentary area fell into the very good quality category.

The crystalline formation of the Apodi-Mossoró basin stood out for the best metric values, with an AG value of 0.88 and a kappa of 0.85. In contrast, regarding the lowest values, the Sertaneja Depression of the Piranhas-Açu basin recorded 0.77 in the kappa index, and the sedimentary portion of Area 3 obtained 0.78 in the AG. The greatest variability in responses was found in the sedimentary portion of Area 3, observed by the presence of lower and upper outliers in both metrics, indicating inconsistencies in the model when distinguishing classes in the randomness of the samples.

Overall accuracy expresses the ratio between the number of correct predictions and the total number of classifications made. In this sense, the model achieved performance above 80% in most areas, except for Area 3, where the value was 78%. The kappa coefficient, in turn, indicated that the reliability of the classifications remained above 75%.

### Confusion matrix of the areas of the Sertaneja Depression

**Fig. 7:**
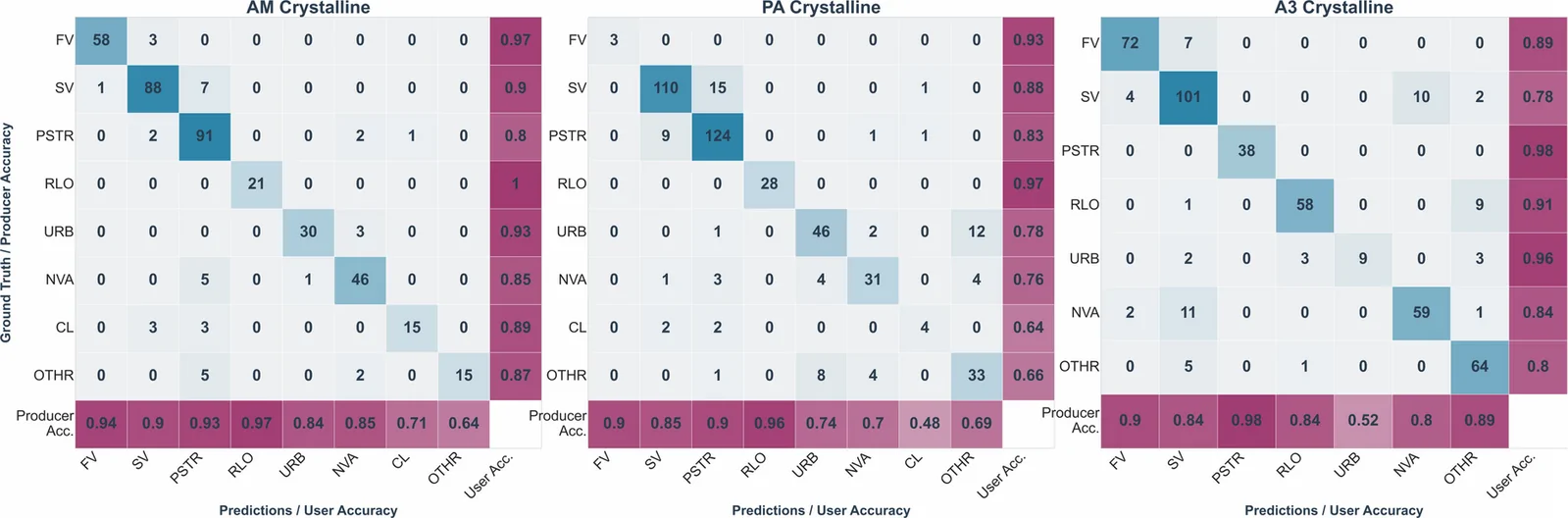
Confusion matrix for areas under crystalline basement. FV, forest vegetation; SV, savanna vegetation; PSTR, pasture; RLO, rivers, lakes, and ocean; URB, urban area; NVA, non-vegetated areas; CL, croplands; OTHR, other users

Corroborating the performance metrics, less variability in confusion was observed in crystalline AM, especially for the river, lake, and ocean class, which was perfectly classified. Other natural areas, such as forest vegetation, savanna vegetation, and non-vegetated areas, obtained an accuracy above 90%. However, in the PA Crystalline area, the model showed a divergence of 12 points between the urban areas class and other uses class, as evidenced by the confusion matrices in Fig. 7. In this region, the other uses class mainly encompasses mineral extraction areas, where exposed soil generates spectral signatures similar to those of urban infrastructure.

In the land use and land cover classifications, confusion between savanna vegetation and pasture is a recurring challenge in the Caatinga biome (Andrade et al., 2021). In this research, it was identified that this occurs especially in areas of geomorphological depression, as found in both PA and AM. Both areas are marked by specific edaphoclimatic characteristics, which promote subtle transitions between natural vegetation formations and anthropized systems, mainly during the deciduous period.

**Fig. 8:**
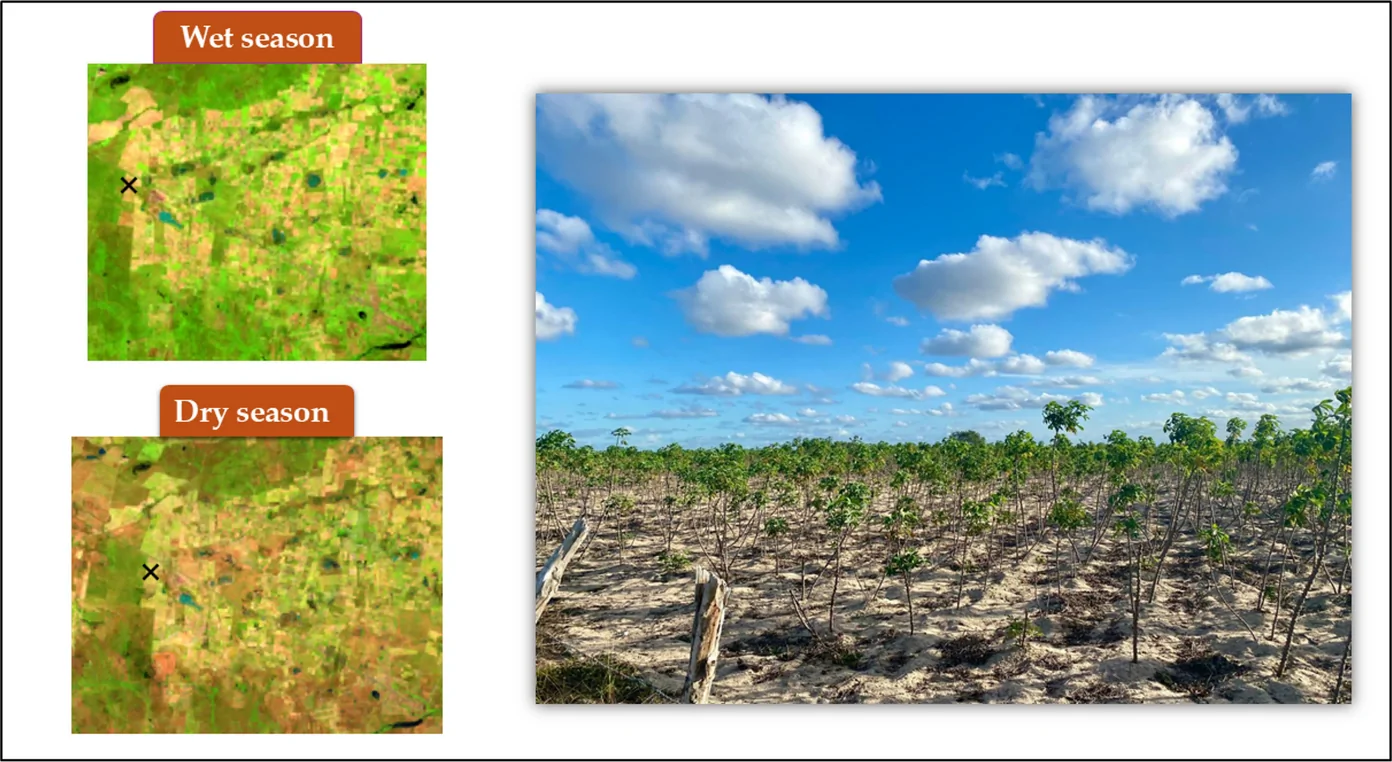
Record of cassava cultivation in the municipality of Serra Caiada (croplands class)

Despite some discrepancies, the statistical values demonstrated high accuracy in classifying vegetated areas in the crystalline areas of the Caatinga biome in Rio Grande do Norte. In all three scenarios, the accuracy results exceeded 90% in forested vegetation and above 84% in savanna cover. Overall, the producer and user accuracy values were considered satisfactory, with overall averages of approximately 0.79 and 0.86, respectively. The croplands category was highlighted in the Sertaneja Depression of Area 3 due to the greater confusion with the pasture class and vice versa, found in 21 points. In this area, changes in land use patterns were observed, mainly regarding agricultural production, where the spectral behavior varies between periods of exposed soil, low vegetation cover, and planting. However, it should be noted that the accuracy for both categories reached an average of 81% (Fig. 8).

Figure 8 shows the record of cassava cultivation in the municipality of Serra Caiada, on a soil with very sandy granulometry (high sand content) on the surface, resulting in high reflectance in the visible and near-infrared bands (Reddy, 2018). In addition, the planting structure presents sparse plants, creating a mixed spectral pattern, which varies between shades of light green, yellow, and pink.

### Confusion matrices of sedimentary areas

In sedimentary areas, similarly to the confusions already pointed out, the main discrepancies occurred between the croplands class and the pasture and savanna vegetation classes in the three contexts analyzed, with greater intensity in A3. This result stems mainly from the spectral similarity in environments of intense anthropic activity, in which management practices and the transition between different uses are frequent, especially between agriculture and livestock farming.

**Fig. 9:**
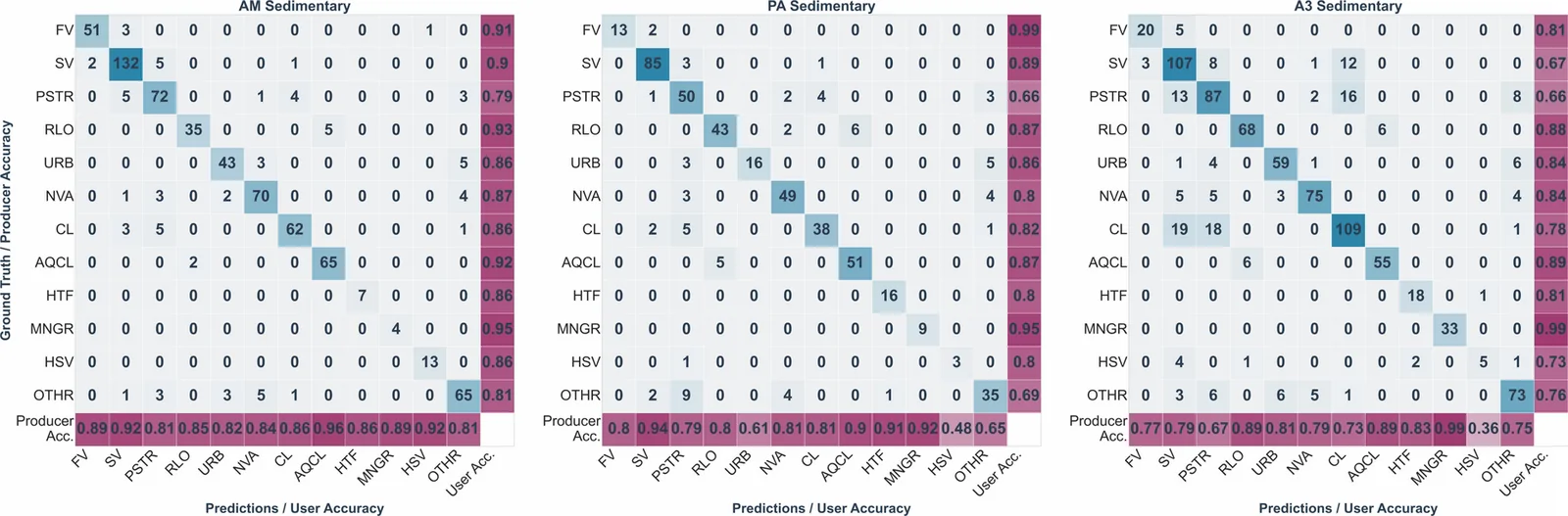
Confusion matrix for areas under the sedimentary basement. FV, forest vegetation; SV, savanna vegetation; PSTR, pasture; RLO, river, lake, and ocean; URB, urban area; NVA, non-vegetated areas; CL, croplands; AQCL, aquaculture; HTF, hypersaline tidal flat; MNGR, mangrove; HSV, herbaceous sandbank vegetation; OTHR, other users

In Area 3, the model showed the greatest discrepancies (Fig. 9). During the photointerpretation phase, this region required additional effort due to the high spectral diversity, the limited availability of field references, and the intense cloud interference in the Landsat-8 images. Located in the eastern portion of the study area, this region presents distinct rainfall characteristics, with higher annual totals, which contribute to the greater cloud cover observed.

In A3, several types of crops were observed, such as papaya, potato, coconut, corn, and sugarcane. According to MapBiomas data (2023), approximately 2320 ha of this area belonged to sugarcane cultivation in 2023. In 2017, the agricultural census of the Brazilian Institute of Geography and Statistics (IBGE) (IBGE 2017) found 38,315 ha dedicated solely to this practice in the state. The spectral behavior of corn and sugarcane is similar, showing greater reflectance in band 6 due to low vegetation cover and soil exposure, both at the beginning of the cycle and during the mechanized harvesting phase, which may cause similarity to pasture cover (Montibeller et al., 2019). Other authors have identified similar mixtures when mapping land uses and land covers in the state of São Paulo, mainly in areas of soybean, sugarcane, and corn cultivation (Shimabukuro et al., 2023).

Despite this, the AP and AU results for most classes were above 65% in all regions, which expresses an adequate quality of the model in predicting untrained pixels. Vegetated terrains (forest and savanna vegetation) obtained AP values between 77 and 94%. Other natural areas, such as non-vegetated areas and rivers, lakes, and ocean, obtained an average AP of 81% and 89%, respectively.

### Analysis of land use and land cover mapping of the Caatinga biome in Rio Grande do Norte

**Fig. 10:**
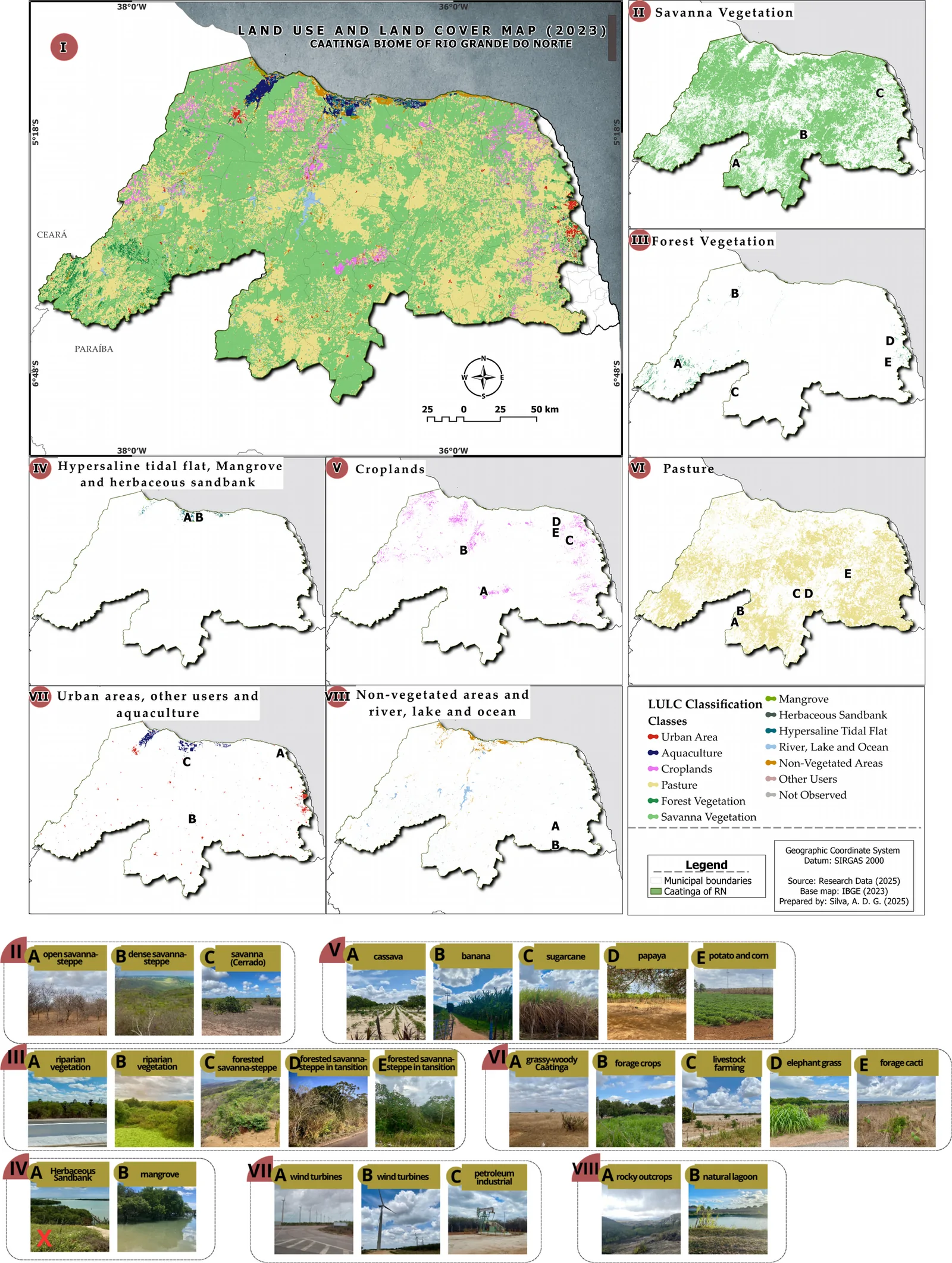
Spatial distribution of the classification of the Caatinga biome in Rio Grande do Norte. (I) LULC of CAARN; (II) savanna vegetation; (III) forest vegetation; (IV) hypersaline tidal flat, mangrove, and herbaceous sandbank; (V) croplands; (VI) pasture; (VII) urban areas, other users, and aquaculture; (VIII) non-vegetated areas and river, lake, and ocean

Figure 10 shows the spatial distribution of each class, highlighting the territorial extent occupied in relative terms (percentages) at the two classification levels. The predominance of native vegetation, whether in its preserved, degraded, or recovering form, is evident, corresponding to ~ 58% of the total area (~ 3 Mha). On the other hand, more than 41% of the Caatinga biome in Rio Grande do Norte corresponds to land use classes associated with anthropogenic influence, such as Anthropic croplands (38%), part of the Non-Agricultural areas (1%), and Other Areas (~ 2%). The quantity of activities conducted by human action mainly envelops agriculture, livestock farming, the ceramic and mining industries, oil exploration, renewable energy projects, as well as aquaculture and urban infrastructure (Fig. 10I).

The savanna vegetation occupies the largest territorial extension within the state, with great representation in the landscape, approximately 2.88 Mha, as shown in Fig. 10II. This is a typical savanna-steppe (Caatinga), marked by arboreal, shrubby, and herbaceous strata, whose density varies according to factors such as soil, climate, relief, and anthropic action. The open steppe vegetation is characterized, above all, by its shrubby/sub-shrubby and herbaceous physiognomy, mainly in the regeneration phase, due to human interference (Fig. 10II A). The dense steppe, commonly found in shrubby-arboreal size, is in plateau areas and low mountain ranges, such as that located in the Borborema Plateau (Fig. 10II B).

In this class, a savanna (biome Cerrado) enclave transitioning to the *Mares de Morros* (sea of hills) morphoclimatic domain was recorded between the municipalities of Pureza and Ceará-Mirim (Fig. 10II C), as reported by Oliveira et al. (2012). An area with a predominance of agricultural uses and wind farm infrastructure was also observed in this savanna enclave. According to Ab’Saber (2003), the concept of an enclave consists of patches of ecosystems typical of other provinces located in different morphoclimatic domains.

With a continuous canopy and tall stature, the forest formation corresponds to a reduced coverage, totaling 37,740.3 ha of the studied area, located mainly in the western and eastern mountain ranges (Fig. 10III), which are the rainiest regions of the state (Lucena et al., 2018; Silva et al., 2022a, b). The forested savanna-steppe, composed of deciduous species, occurs predominantly in areas of mountain escarpments on stony and shallow soils. These characteristics are associated with the particular microclimate of intermediate altimetric elevations, such as in the Martins-Portalegre mountain complex. In addition, remnants of semi-deciduous seasonal forest, composed of species typical of the Atlantic Forest, are found in higher portions, on more permeable and deep soils.

Riparian forests are vegetation formations that accompany the banks of watercourses, playing a fundamental role in protection against erosion, maintaining water quality, and preserving aquatic biodiversity (Junk et al., 2024). In the context of the CAARN, carnauba palm groves stand out, found mainly along the rivers of the western region, such as in the municipalities of Mossoró (Fig. 10III A) and Apodi. The carnauba palm (*Copernicia prunifera*), an endemic palm tree of the Brazilian semi-arid region, is the predominant species in this environment. In addition, a dense arboreal forest is observed on the banks of the Piranhas River, in the municipality of Jardim de Piranhas (Fig. 10III B).

Therefore, in this category, the following were mapped: riparian forests, remnants of seasonal forest in mountain ranges, forested savanna-steppe, documented in the municipality of Martins (Fig. 10III C), and ecological transition areas interspersed with Atlantic Forest species, as in the municipalities of São Gonçalo do Amarante (Fig. 10III D) and Vera Cruz (Fig. 10III E).

In the northern portion of the CAARN, there are transitional estuarine environments in the fluvio-marine plains, composed of a specific vegetation structure. The mangrove ecosystem has two features: mangrove, vegetation composed of arboreal and verdant species; around it is the apicum, an herbaceous vegetation with little expression in the area due to tidal flooding processes (Diniz et al., 2019). In areas with marine influence, there are plant communities, generally on sandy soils, called Restinga (IBGE, 2012). The herbaceous strata are located close to the sea, on beaches, dunes, lagoons, and other fluvio-marine ecosystems. These ecosystems were identified in approximately 16,500 hectares in 2023, being divided into mangrove (4909.4 ha), herbaceous restinga (2479.5 ha), and apicum (9115.9 ha).

As highlighted by the confusion matrices, the restinga (Fig. 10IV A) and apicum vegetation were incorrectly classified, being observed in some points in flooded areas and near the Piranhas-Açu River. The mangrove vegetation (Fig. 10IV B) showed good spatial distribution.

Among the main socioeconomic activities, agriculture, classified as anthropogenic croplands, occupies around 135,000 ha of the Caatinga biome. Extensive areas of cassava and cashew cultivation stand out in the Serra de Santana (Fig. 9V A); banana plantations in Ipanguaçu (Fig. 9V B); coconut, corn, and sugarcane in Pureza (Fig. 9V C); and papaya, potato and corn in São Miguel do Gostoso (Fig. 9V D and V E).

In the pasture class, herbaceous species predominate, encompassing both native pastures and cultivated areas for direct or indirect animal feed. These environments are widely used for cattle grazing, which marks the grassy-woody Caatinga aspect. In the municipality of Jardim de Piranhas (Fig. 10VI A), extensive areas occupied by sparse herbaceous vegetation were identified, with the presence of *Agrostis pourretii* and some cacti. Also in this location, forage crops were identified near the river floodplains (Fig. 9VI B). According to Silva, Souza, and Lucena (2023), open vegetation is the most predominant characteristic in the aforementioned municipality.

Figure 10VI C shows an area with low vegetation cover density, used for livestock, in the rural area of Lagoa Nova. In addition to these landscapes, there is the cultivation of forage crops, such as elephant grass (Fig. 10VI D) and forage cacti (abandoned area Fig. 10VI E). These practices are common due to their high productivity and efficiency in providing food for livestock. According to the results, pastures total approximately 1.77 Mha in the region.

The other uses class encompasses industrial and commercial areas, communication networks, mineral extraction, oil, and renewable energies (wind and solar farms), occupying 21,376.6 ha. Solar and wind energy stand out, transforming not only the energy landscape but also coastal and mountainous landscapes and vegetation cover, in particular. At the level of non-agricultural anthropic areas, urban infrastructure and aquaculture account for 65,895 ha (1.3% of the total), with 30,772.6 ha corresponding to medium and high-density urban areas.

Figure 10VII shows the distribution of these classes. The analysis of the classification shows that the model was able to identify not only dense urban areas but also small, dispersed communities. Areas with high urban planning are locations with complete road systems, paving, and urban afforestation; in contrast, areas with low planning have spaces devoid of urban complexity (São Paulo, 2016). Despite this performance, pixels classified as other uses were observed in some more urbanely planned sectors, a result consistent with the inconsistencies already highlighted by the evaluation metrics. A linear pattern is also observed in this class, mainly associated with the presence of wind farms and oil complexes. Representative examples can be seen in the municipality of Touros (Fig. 10VII A), where several wind turbines are in operation in a transition area of the savanna enclave (biome Cerrado), and in Serra de Santana (Fig. 10VII B), an important wind power generation hub in the state. Figure 10VII shows a mechanical pumping platform near the municipality of Alto dos Rodrigues.

The spatial distribution of the aquaculture class (Fig. 10VII) revealed a pattern consistent with its actual distribution. However, the precision values (AP = 0.46 and AU = 0.45) indicate challenges in differentiating it from the Water class. During the reinterpretation stage, adjustments to the samples were made to reduce this confusion, resulting in a significant improvement in the final classification.

Other natural surfaces, such as non-vegetated areas, include rocky outcrops, coastal dunes, and sandbanks in riverbeds and banks, totaling 48,800 ha (0.96% of CAARN). Figure 10VIII A shows the distribution of these environments, with an example registered in the municipality of Serra de São Bento. Water bodies occupy 60,511.61 ha of the total area, represented by rivers, lakes, lagoons, reservoirs, and ocean waters along the northern coast. Among them, natural lagoons identified in the Sertaneja Depression of Area 3, in the municipality of Januário Cicco, stand out (Fig. 10VIII B).

### Analysis of mappings in geomorphological compartments

The compartmentalization of the Caatinga biome in Rio Grande do Norte into relief structures resulted in two areas: (1) one under crystalline basement, corresponding to approximately 30,422 km^2^ of the area, and (2) one under sedimentary basement, with an extension of 20,280 km^2^. The analysis of the distribution of classes in the geoenvironments shows the predominance of shrubby-arboreal savanna-steppe (savanna vegetation), which occupies approximately 1.6 Mha in the crystalline base and 1.2 Mha in the sedimentary base. Table 2 presents the proportions of each class at different hierarchical levels.

**Table 2:** Absolute and relative distribution of the area of level II classes by geomorphological compartments

Usage and coverage classes	Geomorphological compartments					
	Crystalline			Sedimentary		
	Area (ha)	Area (km^2^)	% of the total	Area (ha)	Area (km^2^)	% of the total
Forest vegetation	19,943.15	199.73	0.39	17,799.83	178	0.35
Savanna vegetation	1,640,344.49	16,393.44	32.32	1,238,860	12,384.65	24.40
Mangrove	-	-	-	4,908.06	49.08	0.10
Herbaceous coastal vegetation	-	-	-	2,479.58	24.8	0.05
Apicum	-	-	-	9.086.77	90.87	0.18
Croplands	17,157.68	169.78	0.34	117,555.26	1,175.55	2.32
Pasture	1,283,599.07	12,809.19	25.29	489,340.74	4,893.41	9.64
Urban area	10,674.84	106.22	0.21	20,151.45	201.51	0.40
Non-vegetated area	7,967.71	79.7	0.16	40,830.58	408.36	0.81
Rivers, lakes, and ocean	44,306.86	443.11	0.87	16,204.75	162.04	0.32
Aquaculture	-	-	-	35,122.46	351.22	0.70
Other uses	3,328.44	33.3	0.07	18,046.5	180.46	0.36
Not observed	13,108.58	131.09	0.26	17,205.22	172.05	0.34

The pasture category covers significant areas in both compartments, with a higher concentration in the Sertaneja Depression. This scenario can be explained, in part, by the conversion of natural areas to agricultural and livestock uses, a process that results in a landscape marked by mixed-use patterns and low vegetation cover. However, vegetation density can define these results, as areas of open and hyperxerophilous savanna-steppe are more recurrent in these environments. Research shows that 51.75% of the total area of the Seridó River basin, partly located in RN and belonging to the influence of the Piranhas-Açu basin, is composed of this type of Caatinga (Oliveira et al., 2022). In the livestock sector, as pointed out by the agricultural census of IBGE (2017), the microregions of Seridó showed greater prominence, driven mainly by the expansion of milk production.

Despite being smaller in area, agricultural production is more pronounced in sedimentary terrains, 117,555.26 ha (2.32% of the total area), while in the crystalline base, this value is approximately 17,000 hectares (0.34%/total). This predominance may be related to the particularities of these terrains, such as the presence of deeper soils and their lower slope (Jacomine et al., 1971). The sedimentary compartment provides petroleum activity and the exploration of raw materials for the manufacture of cement, calcium oxide, etc., which are included in the other classes, in addition to renewable energy ventures, which together cover an approximate area of 18,000 ha in the sedimentary compartment. Aquaculture covers approximately 35,000 hectares of the study area. Thus, in the CAARN, savanna vegetation predominated, followed by pasture and agricultural land use, accounting for 56.72%, 34.93%, and 2.66%, respectively.

### Importance of variables

**Fig. 11:**
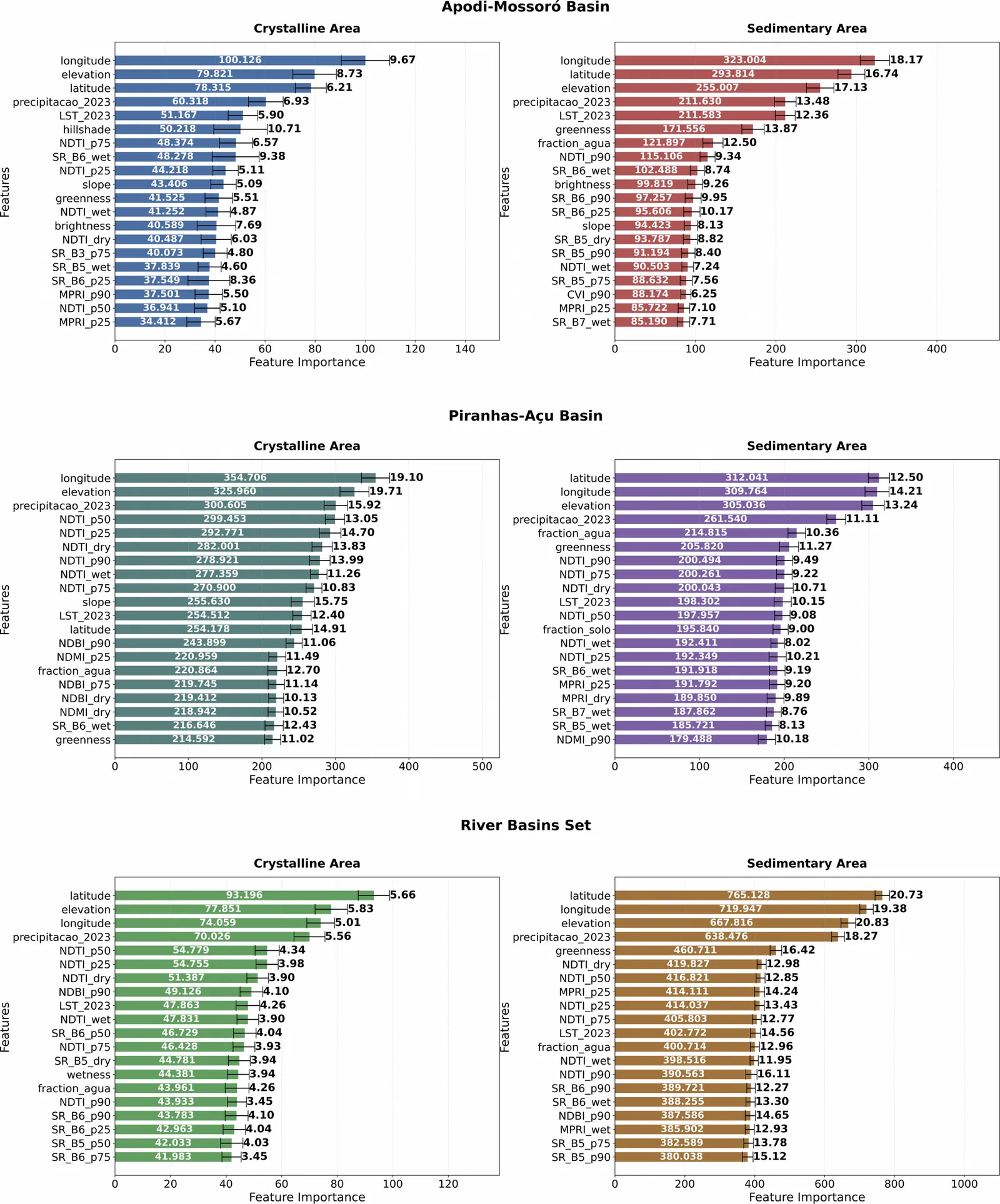
Ranking of the 20 most relevant covariates in the CAARN usage and coverage classification

The 153 layers used in the classification were organized according to their level of importance for each fragmentation of the study area. However, Fig. 11 shows the ranking of only the 20 most relevant covariates. The results indicate that, in all geomorphological compartments, the most important variables were those related to spatial position (longitude and latitude), topographic attributes (altitude), and climatic conditions (precipitation and surface temperature). These findings highlight the relevance of integrating physical-environmental aspects in the characterization of spatial targets in different contexts of the CAARN.

Among the biophysical indices analyzed, NDTI and its variations (percentiles and wet and dry periods) stood out in all sub-areas, due to their ability to estimate crop residue cover and indicate cultivation intensity, constituting an essential tool for monitoring agricultural practices. NDBI, aimed at identifying built-up and urbanized areas (Gao, 1996), and MPRI, used for soil and vegetation analysis (Yang et al., 2008), also proved to be relevant attributes.

In the analysis of water classes, the water fraction image (fraction_agua) and NDMI showed greater relevance than the mNDWI and NDWI spectral indices. To differentiate vegetated targets from exposed areas, the transformed greenness and brightness bands (TCT), as well as the NDBI and MPRI indices, stood out. The spectral bands of the near-infrared (NIR) and short-wave infrared (SWIR1) channels also played a significant role. The NIR band (B5) is used to detect photosynthetic activity, since it is the spectral range with the highest reflectance, relating to the presence, leaf structure, and density of species. Regarding the SWIR1 band (B6), it is sensitive to identifying water stress in plants.

### Comparison between methods of area fragmentation

The Apodi-Mossoró and Piranhas-Açu river basins, in Rio Grande do Norte, are divided into crystalline and sedimentary geoenvironments. Based on this observation, a comparison was made between the mapping obtained with this approach and the traditional classification method, based on the complete delimitation of the river basins, named AM Complete and PA Complete, respectively. Both areas were processed with the same set of samples and subjected to adjustment of the number of trees using the random forest algorithm.

**Fig. 12:**
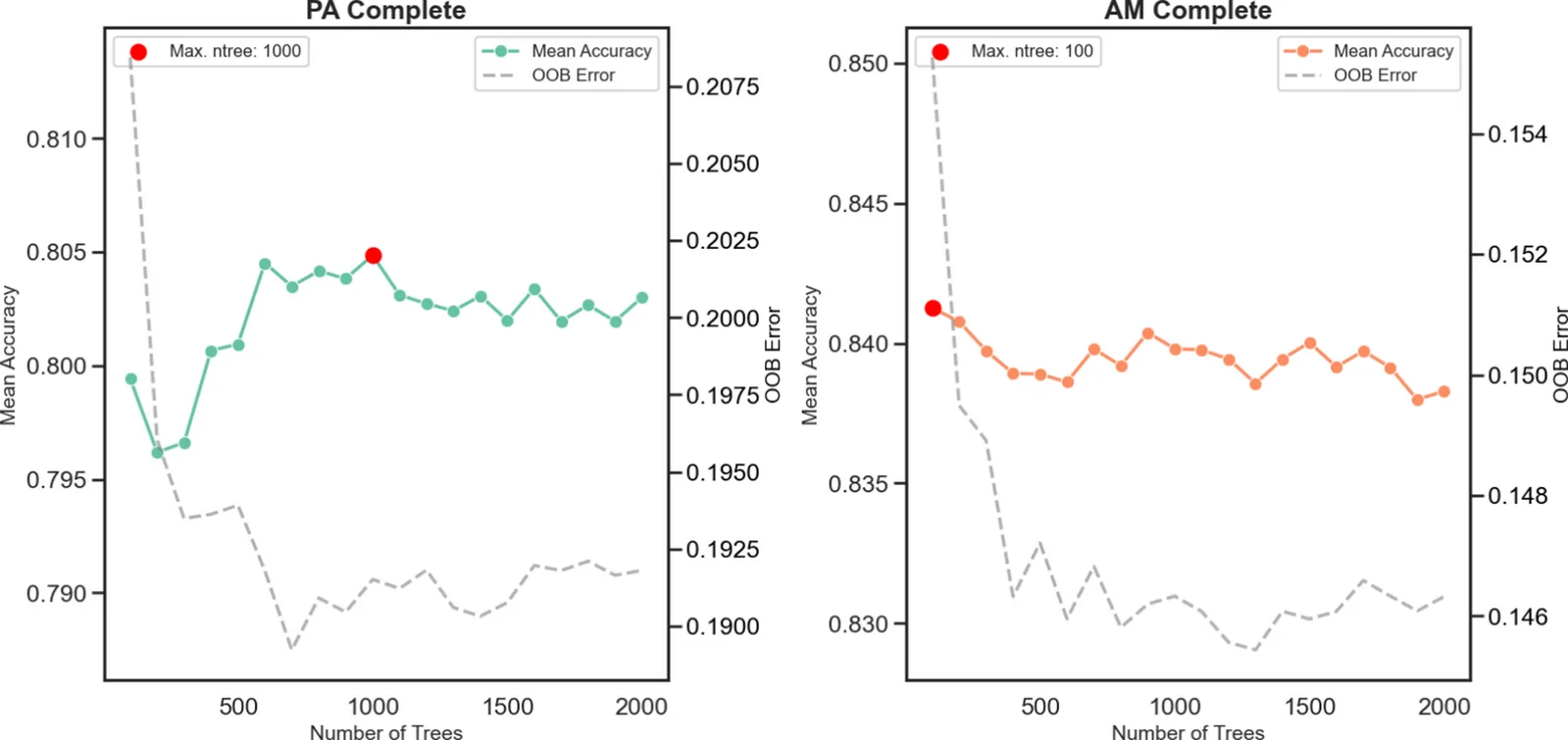
OOB and ntree results for the Piranhas-Açu and Apodi-Mossoró river basins

Following the preliminary methodological process, the algorithm was applied to find the best number for the number of trees parameter based on the global accuracy metric. The results indicated that the ideal configuration of the ntree parameter showed similar behavior to that verified in previous analyses. In the Piranhas-Açu drainage basin, the best performance was obtained with ntree = 1000, associated with low OOB error values. For the Apodi-Mossoró drainage basin, the highest accuracy did not coincide with the lowest OOB; in this case, the value ntree = 700 was chosen, as illustrated in Fig. 12.

**Fig. 13:**
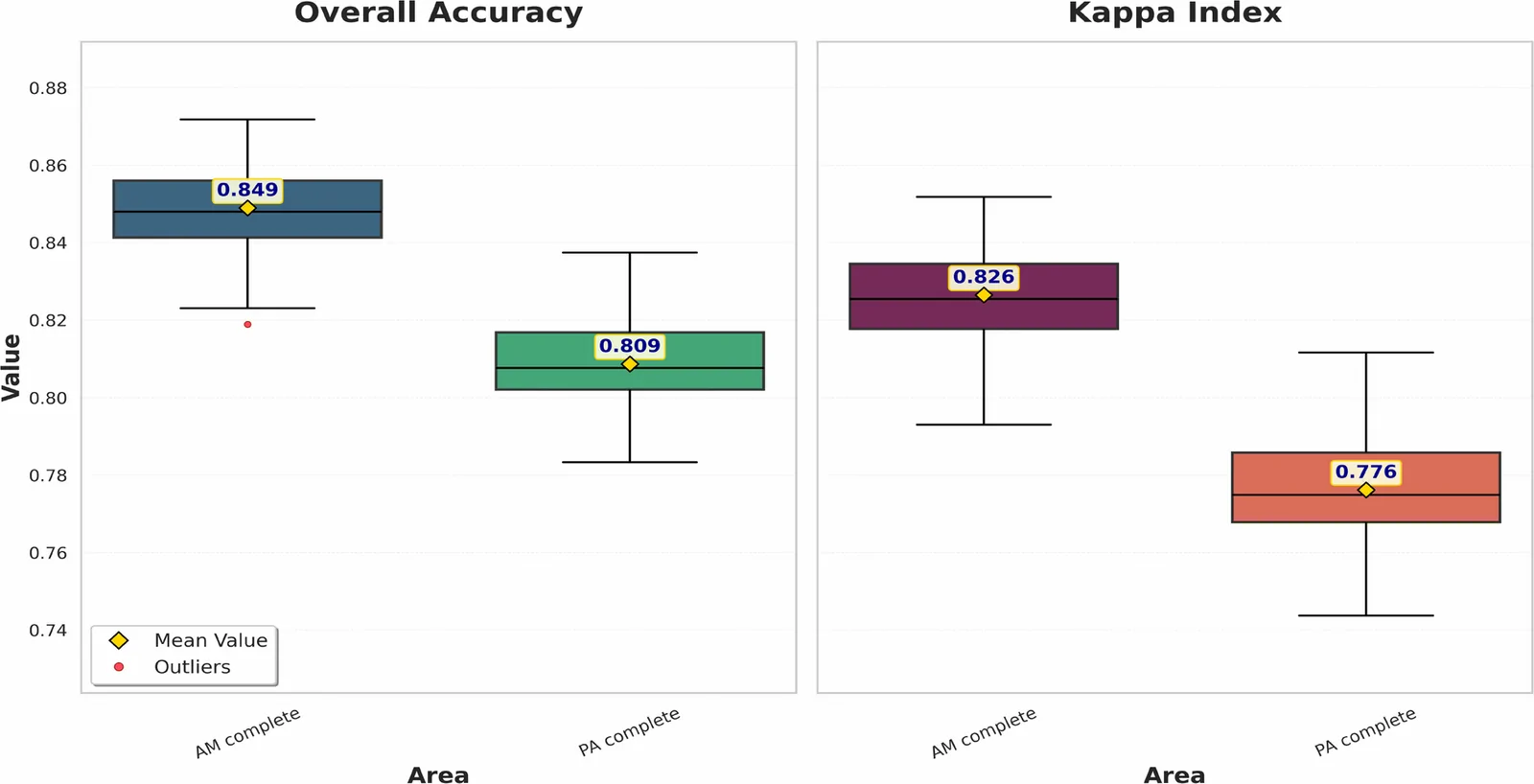
Overall accuracy values and kappa index for the Piranhas-Açu and Apodi-Mossoró river basins

Regarding the AG performance metrics and kappa index, presented in Fig. 13, it is observed that, in general, the machine learning model achieved an accuracy greater than 80% in the two analyzed drainage basins. The kappa index indicated a “very strong” agreement for the AM Complete and a “very good” rating for the PA Complete. These results are consistent with the values obtained in the approach based on relief macrostructures.

**Fig. 14:**
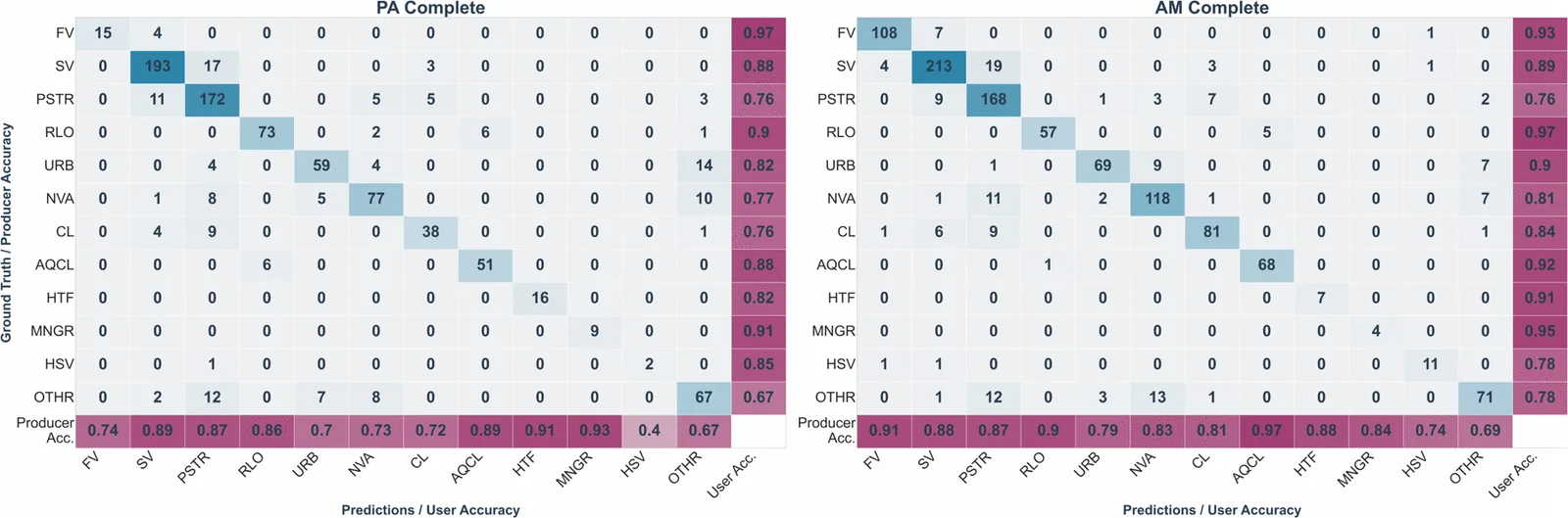
Confusion matrix for the Piranhas-Açu and Apodi-Mossoró river basins. FV, forest vegetation; SV, savanna vegetation; PSTR, pasture; RLO, river, lake, and ocean; URB, urban area; NVA, non-vegetated areas; CL, croplands; AQCL, aquaculture; HTF, hypersaline tidal flat; MNGR, mangrove; HSV, herbaceous sandbank vegetation; OTHR, other users

Despite the statistical similarity between the methods, discrepancies were observed in the spatial distribution of the classes. In the method using the complete areas of the drainage basins, the confusions already pointed out previously showed a significant increase compared to the approach by geomorphological domains. This means that the errors in distinguishing between artificial typologies became more evident, such as urban areas and other uses, and pasture and non-vegetated areas (Fig. 14).

**Fig. 15:**
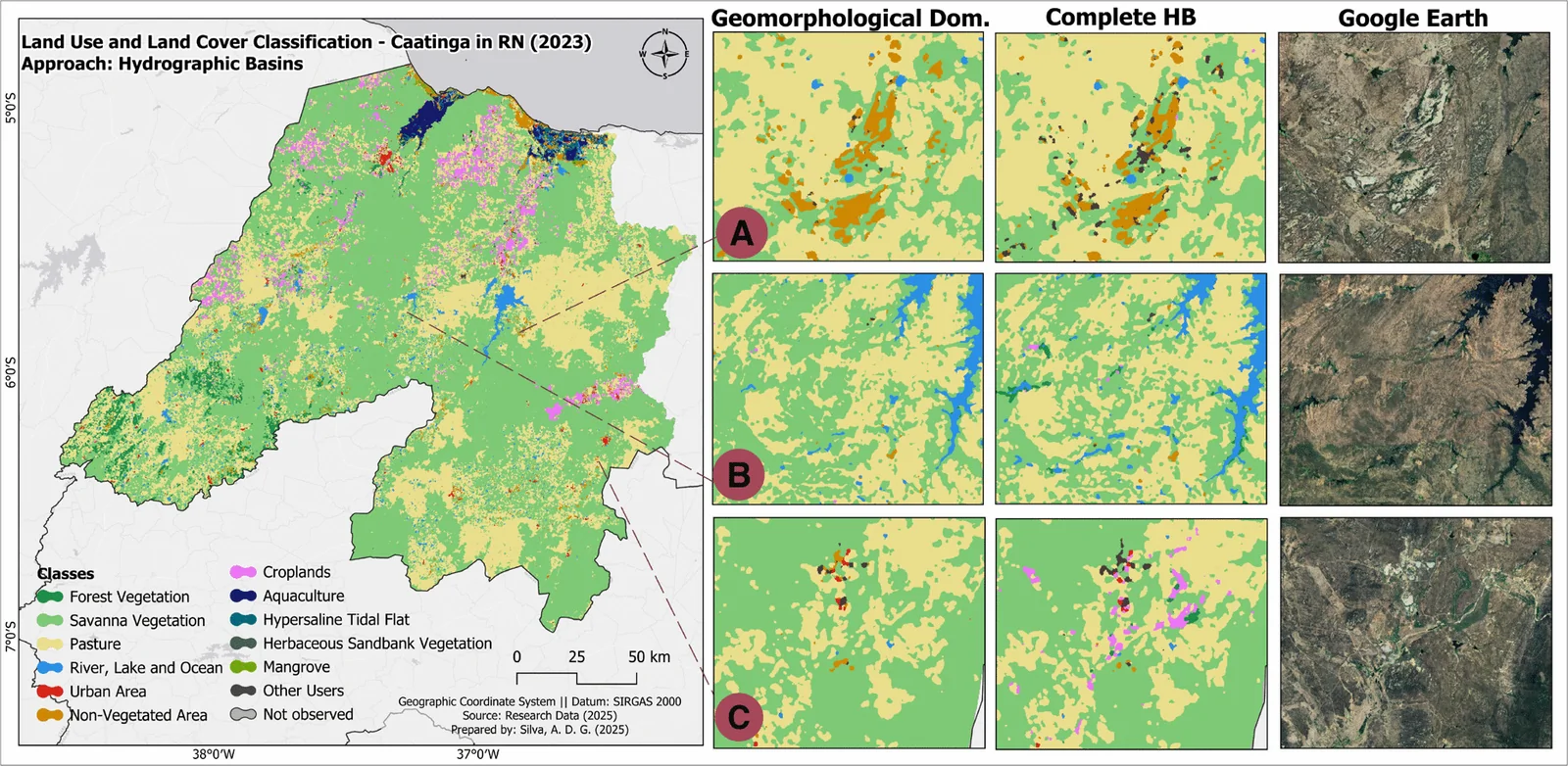
Comparisons of land use and land cover classification using a drainage basin approach versus separation by geomorphological domains in each drainage basin

Upon examining the spatial arrangement, it is observed that the model attempts to minimize the associated uncertainty by making general groupings; thus, land use classes, such as urban areas and other uses, and the non-vegetated area class, were aggregated into other uses (Fig. 15). This generalization represents a challenge, as it limits the ability to adequately capture and differentiate the various forms of human occupation. For better visualization, the other uses class was highlighted in a dark tone.

Figure 15A illustrates an example of this inconsistency in an area of exposed rock located in the Piranhas-Açu basin. The error became evident when compared to the results of the scenario structured from the relief compartments, which demonstrated greater correspondence with the samples and better representation of the real terrain conditions. Furthermore, it was difficult to classify surfaces with homogeneous reflectance, as occurs in extensive dunes and sandy deposits of riverbeds.

In the case of vegetation cover, the modeling showed similar behavior between classes, except for the forest vegetation category. Figure 15B shows the occurrence of pixels located near water bodies in the Apodi-Mossoró basin, where the availability of water throughout the year favors high levels of photosynthetic activity. This condition allows for the development of more vigorous vegetation, even during periods of drought, altering the spectral signature and generating ambiguities in the classification process. The forest vegetation class was exclusively delimited to represent riparian forests, composed of tree formations with a continuous canopy.

The difficulty of photointerpretation in certain croplands due to the complex pixel mosaic has already been extensively discussed in this manuscript. As observed in Fig. 15C, this approach expanded the croplands in the crystalline environment, not reflecting the real conditions of the Piranhas-Açu Basin. These are locations associated with water drainage, which favors high vegetation vigor throughout the year, a condition spectrally similar to the irrigated croplands observed in the sedimentary portion.

## Discussion

Spatial information plays an essential role in monitoring and planning environmental conditions, especially vegetation cover. In this research, the model designed for land use and land cover classification of the Caatinga biome in Rio Grande do Norte for the year 2023, using the RF algorithm processed in GEE, proved to be adequate and efficient for the dimensionality and variety of the data. This means that the combination of techniques for adjusting the number of trees, the proposed area subdivision, the multiple runs of the algorithm, and the covariates provided opportunities for excellent analyses.

Relief influences both environmental variables, such as soil type and predominant rocks, water availability, vegetation cover, and socioeconomic land uses (Bulliqi et al., 2020; Reed et al., 2009); therefore, variations in spectral properties in optical products are noted. Thus, used as a grouping criterion, it contributes to reducing omission and commission errors, according to the research results. In the Sertaneja depression, spatialization in the classification by hydrographic basins showed that the agriculture typology was overestimated in water drainage areas and the other anthropic uses class in urban areas and non-vegetated areas. Thus, the hydrographic basin, when considered in its entirety, encompasses diverse environments with distinct geomorphological spectral characteristics, leading to more errors.

Regarding hyperparameters, the variation in the optimal values of ntree is consistent with the theory that the RF’s generalization error converges in a non-monotonic manner; that is, the gain in accuracy beyond a data-dependent threshold becomes negligible (Breiman, 2001; Ming et al., 2016). This threshold depends on the heterogeneity and separability of the classes in the training data (Svoboda et al., 2022), which explains why more spectrally heterogeneous regions, such as the crystalline PA, require a larger set of trees for stabilization. This finding reinforces the need to tune the hyperparameters specifically for each environment.

The performance of the model obtained in this study is consistent with similar RF-based applications for classifying surface targets in semi-arid environments. In the Upper Tezeke Basin in Ethiopia, classification results were satisfactory despite the topographic and climatic complexity (Fentaw & Abegaz, 2024). In the sertão region of Ceará, Brazil, the landscape is heterogeneous and the vegetation is dependent on seasonal climate patterns (Marzulo Maia Martins et al., 2024). In northern Mozambique, RF performed satisfactorily in classifying six classes, including dense and deciduous vegetation (Macarringue et al., 2023).

Analysis of the uncertainty map of the iterations reveals a direct relationship between performance metrics and model uncertainty patterns (see Fig. 5). Areas with greater classification difficulties, such as those with anthropogenic use and coastal vegetation, also showed the highest uncertainty index. On the other hand, regions with consolidated natural cover and homogeneous vegetation demonstrated low levels of uncertainty, corroborating the high accuracy indices observed in the forest vegetation and water bodies classes.

The environmental variables used to map the variability of spatial typologies, such as estimated precipitation, surface temperature, morphometric characteristics, and geographic location, proved relevant in all scenarios studied. Geographic location and altitude are considered geographic factors of climate and are conditioning factors that can shape the behavior of precipitation and temperature, influencing soil conditions and, consequently, the size and density of vegetation, as well as the types and intensity of anthropogenic activities (de Araújo Filho et al., 2022; Correia Filho et al., 2019; Fritzsons et al., 2015; Reed et al., 2009). The amount and spatial distribution of rainfall influence the cyclical growth/flowering process and the density of seasonally dry vegetation, thereby improving the accuracy of LULC mapping (Júnior et al., 2022).

To investigate the direct implications of the variables on the interpretability of the different classes, the importance per class (mean decrease in accuracy—MDA) was calculated for the six study areas (figure). The results reveal that spatial and topographic variables are crucial for the Forest, Apicum, Restinga, Aquaculture, Agriculture, Bare Land, and Other Land Use classes. Forest vegetation is distributed primarily in the western portion of the state, where topographic and climatic conditions favor its occurrence. Apicum, Restinga, and Aquaculture are restricted to sedimentary areas, such as the northern coastal strip and a small occurrence in the east. Agriculture is also distributed in clusters because it depends on irrigated areas and alluvial soils near rivers and reservoirs, with the highest concentration in sedimentary areas. In contrast, for the River, Lake, and Ocean; Savanna; Urban; and Mangrove classes, these variables are not significant, and spectral and biophysical predictors remain the primary factors.

Other studies highlight the importance of auxiliary variables, such as topography (elevation, slope) (Riley et al., 2016; Shandu et al., 2026), tasseled cap (Sovann et al., 2025), geographic coordinates (Milà et al., 2024; Riley et al., 2016; Sparey et al., 2024), temperature, and precipitation (Riley et al., 2016; Sparey et al., 2024), the latter being especially important for improving land use and land cover classification in seasonally dry tropical forests (De Paula Sousa Sousa Júnior et al., 2022).

Latitude directly influences the intensity and duration of the solar radiation received at each location. It corresponds to the distance of a given location from the equator; thus, as latitude decreases, solar incidence increases, leading to higher temperatures. Regarding apparent surface temperature, it is calculated from the radiation emitted by Earth’s targets (Forooshani et al., 2023), proportionally related to land cover conditions. Therefore, areas with exposed soil, vegetation under pressure, and anthropogenic uses tend to have higher surface temperatures, while the presence of soil moisture and greater vegetation cover reduces LST values, highly relevant for identifying pasture. Rees et al. (2024) have identified that high LST values correspond to urban areas and bare lands, whereas low LST values were observed in areas with dense vegetation cover.

Research indicates that spatial proxies can improve the prediction of environmental variables when the phenomenon exhibits spatial autocorrelation (Milà et al., 2024); however, these highly autocorrelated coordinates may cause the RF to merely replicate the spatial patterns of the training data, thereby improving the model’s performance but reducing its generalization ability (Meyer et al., 2019).

The territory of the state of Rio Grande do Norte is formed by the structural bases of reliefs under crystalline basement and under sedimentary formations, which is directly reflected in the grouping of spectral response patterns in satellite images. In general, sedimentary environments are frequently formed by rocks such as sandstones and limestones, and their soils have a greater capacity for water retention and are deep, allowing for the insertion of agricultural mechanization and larger-scale agriculture. In addition, their surface colors are lighter due to the low levels of hematite (iron oxide) (Jacomine et al., 1971). Soils under crystalline basement are marked by the relatively greater presence of iron oxides, such as hematite and goethite, giving them reddish and yellowish colors, and are shallower and stonier, hindering agricultural production (Gusmão Silva et al., 2019; de Souza et al., 2021).

**Fig. 16:**
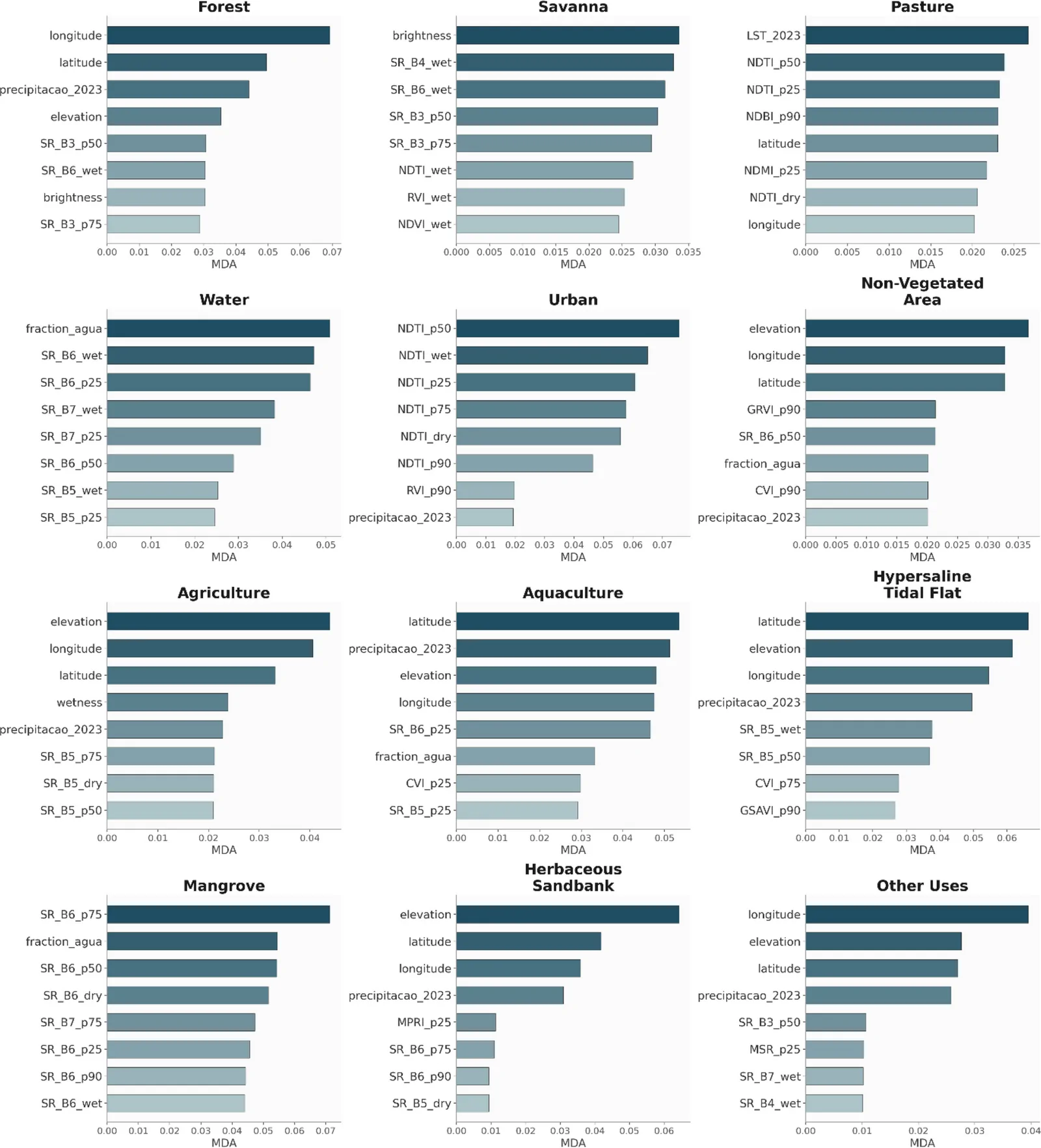
Ranking of the eight most relevant covariates in the CAARN by classes

Consequently, the agricultural performance of the CAARN presents a dichotomy between modern agriculture and family-managed subsistence farming (Silva & Troleis, 2018). The former refers to large-scale production focused on irrigated fruit growing, mainly destined for export, highlighting the Açu-Mossoró irrigated fruit growing hub, fully integrated into the sedimentary formation, favored by investments in technology, irrigation infrastructure, and external markets, due to the water availability of the large Piranhas-Açu and Apodi-Mossoró river basins. This situation has been restructuring the economy, in the face of agricultural advancement (Pereira et al., 2009), and highlighting social issues (Morais et al., 2023). In contrast, in the Sertaneja Depression (crystalline basement), family-managed subsistence farming faces disadvantages related to both climate and soil type for the cultivation of low-productivity corn, beans, and cassava, due to greater dependence on rainfall (Vale et al., 2020). In the Seridó microregion, for example, there is a stronger link between Family farming and mineral extraction (Santos et al., 2022). This geographic area has been known for records of scheelite mineral exploration since the 1940 s (Da Silva et al., 2022a, b). Studies are still being conducted to assess the environmental effects resulting from this form of natural resource exploitation (Garcia et al., 2025). The dichotomy in agricultural distribution is consistent with the high significance of longitude and elevation identified for the reference class (see Fig. 16), reflecting the region’s geological and socioeconomic gradient.

In the overall design of the economic activities of the state, the geological structure characterized as sedimentary provides ceramic production, responsible for extracting firewood and clay (Lopes-Nunes et al., 2022); the complex of oil and gas extraction; and exploration of raw materials for the manufacture of cement, calcium oxide, etc., in the north-central portion. The northern coast has salt production and shrimp farming, which involves the deforestation of mangrove vegetation (Beltrão-Sabadía et al., 2024). This panorama highlights the contribution of the physical environment as support for the analysis of spatial organization, such as the type of geological basement, as it triggers an environment for the configuration of agricultural activities in the CAARN.

The greater heterogeneity of socioeconomic activities, as the analysis extends to the coast of the CAARN, is reflected in the complexity of analyzing classes and spectral confusions, especially in croplands. In parallel, in the Sertaneja Depression, most agricultural production is concentrated in small rural properties (Locatel, 2018), sometimes irregularly arranged, with dispersed cultivation areas interspersed with native vegetation or other forms of land use, such as livestock farming. This fragmentation affects the process of detecting and discriminating planting, as variations in texture and spectral characteristics, especially colors, can overlap.

Regarding the natural vegetation cover, in seasonally dry forests like the Caatinga, the combination of edaphoclimatic aspects provides distinct characteristics, both in the vertical stratum of the plant and in the density of spatial cover. Specifically, water availability and soil type lead to a dense horizontal vegetation structure; conversely, areas with low rainfall tend to have sparse vegetation cover (De Almeida et al., 2019; Moro et al., 2016).

Areas of natural vegetation cover the largest territorial area of the CAARN, especially the savanna formation, in 2023, varying in density and size depending on the environment. In soils of sedimentary environments and mountain slopes, it is organized more continuously and compactly, that is, the dense shrubby savanna-steppe is more representative. In addition, the mountains contribute with a greater variety of species and plant strata, with research pointing to remnants of forest formation in the mountain complex located in the southwest of the state, for example in Queiroz et al. (2021) who carried out a floristic analysis in Serrinha dos Pintos.

**Fig. 17:**
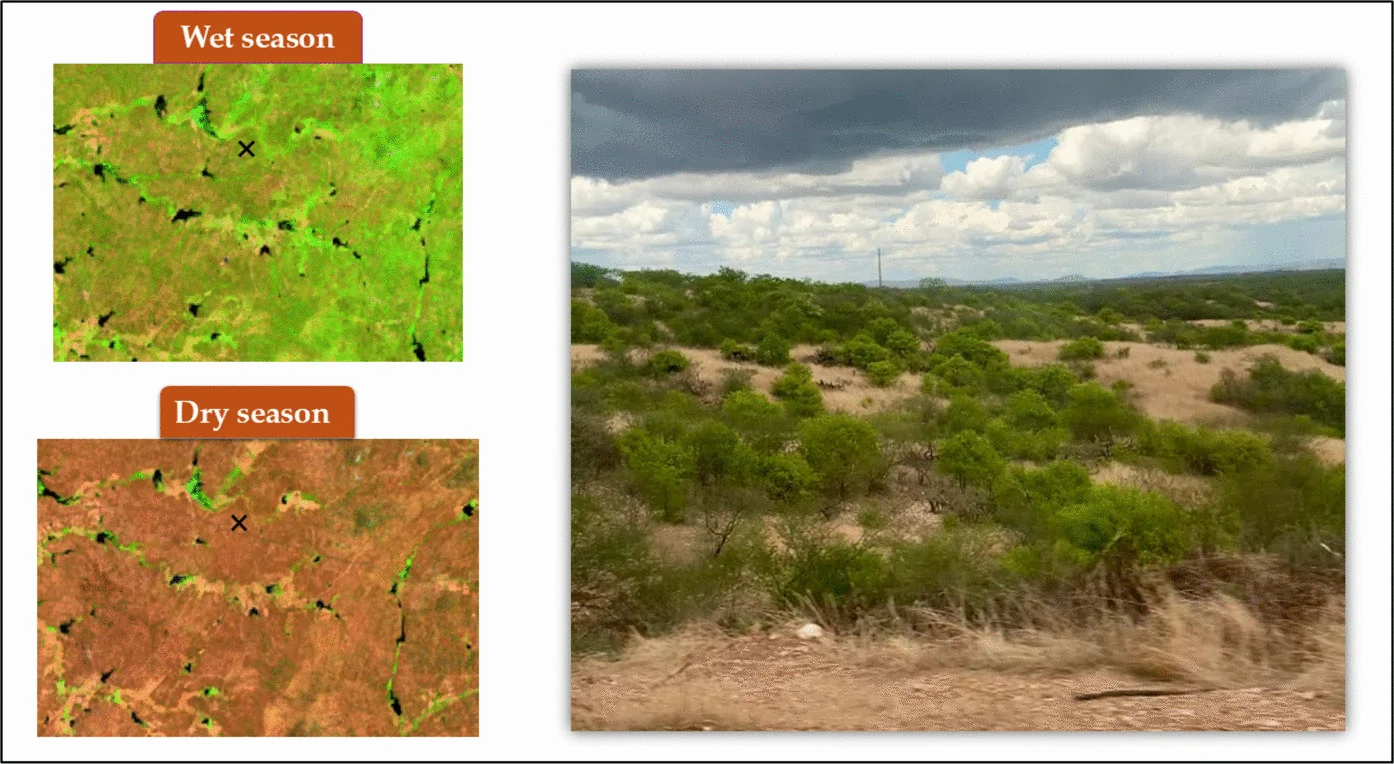
Shrubby savanna-steppe with an open horizontal structure

In the crystalline basement, the shrubby vegetation presents a horizontal structure with a high degree of openness and easily transitions to sub-shrubby strata, resembling a park and grassy-woody physiognomy. A record made in the municipality of Acari exemplifies this landscape of shrubby species spatially interspersed with herbaceous plants, such as the panasco grass (*Aristida setifolia*), on a gently undulating relief (Fig. 17). The presence of vegetation during the rainy season is noticeable by the shades of green in the image; however, interspersed with uncovered soil in a pinkish hue. During the dry season, the loss of foliage results in the predominance of earthy tones, similar to the exposed soil, consequently highlighting the confusion between the predominant vegetation classes and pasture areas. Based on the results, the savanna vegetation class was confused with pasture in 45 samples, with 29 samples being in the Sertaneja Depression. These observations were also discussed in the work by Andrade et al. (2021) when conducting the mapping for the state of Paraiba.

This environment is located in an area susceptible to desertification. The high degree of degradation in this area is due to both natural characteristics, such as shallow and stony soil and low water retention capacity, as well as the removal of vegetation cover for clay extraction and overgrazing. It is worth noting that this area has the most arid climate type in the state (Silva et al., 2022a, b). Overgrazing reduces growth and geographic distribution, affecting the regeneration capacity of the vegetation (Araújo Filho 2013). The pasture class found in the research revealed that more than 25% of the total is concentrated in the crystalline base, while about 10% of the total is in sedimentary areas.

Vegetation is the natural protection of the soil against the erosive action of rain and wind, protecting against the loss of chemical and physical qualities (de Araújo Filho et al., 2022) and against water runoff and greater loss of soil moisture through evaporation. This protection helps maintain thermal control, mitigating the effects of climate change (Lucena et al., 2021), biodiversity (Oliveira et al., 2019), and cultural and ecosystem services (Moreira et al., 2019; Refati et al., 2023), which are important in seasonally dry environments. A trend of reduction in vegetation categories in the Caatinga biome between 1985 and 2019 was detected by Franca Rocha et al. (2024), with 11% for savanna-type vegetation and 8% for forest formation. In parallel, there was an increase in non-natural areas (27%), with a predominance of pasture (25%).

Silva et al. (2025), studying climate projections on Caatinga plants up to 2100, discovered that a variety of invasive species, particularly non-woody plants such as grasses, are more likely to persist and expand under warmer and drier future climate conditions. In contrast, tree species are predicted to decline in this biome, considering the climate scenarios predicted for the end of the century.

## Conclusions

Considering the methodological procedures employed, the results achieved, and the analyses performed, it is concluded that:

i) Adjusting the number of trees was an important step in enhancing the performance of the models. The RF algorithm achieved satisfactory performance for the classification of 12 typologies, obtaining an average accuracy of 83% and a kappa value of 0.8.
ii) The predicted spatial distribution showed greater reliability compared to real data in areas of natural cover, such as natural vegetation (forest vegetation and savanna vegetation) and water bodies (rivers, lakes, and ocean), although some inaccuracies occurred in the herbaceous restinga and apicum classes. The spatial distribution of the pasture areas, croplands, and aquaculture classes was adequate. The category encompassing other anthropogenic activities (other uses), non-vegetated areas, and urban areas showed positive results, despite minor overlaps, which is expected given the spectral similarity between these classes.
iii) An analytical look at the division of the Caatinga biome in Rio Grande do Norte into geomorphological compartments allowed us to cross-reference the relationship between relief and vegetation patterns and land uses. In the Sertaneja Depression, a greater proportion of sparser vegetation and activities related to livestock farming and mining were observed. Under the sedimentary basement, the vegetation cover shows a lower degree of openness, and land use is mainly linked to irrigated fruit cultivation. Regarding the regions of high-altitude relief and low mountain ranges, the surface vegetation cover presents a closed structure, with a continuous canopy.
iv) The research showed that environmental variables were the most relevant in all environments, such as spatial position (longitude and latitude), topography (altitude), and climate (precipitation and surface temperature), indicating that physical and climatic factors play a determining role in characterizing the Caatinga biome.
v) Comparative analyses conducted between mapping approaches based on dividing areas by geomorphological domains and by hydrographic basins highlight the complexity involved in the digital classification of extensive areas with heterogeneous spatial patterns. The results obtained do not conclude the discussion on the subject but contribute to a deeper understanding of the influences of environmental variables on the spatial behavior of the Caatinga vegetation. Therefore, this study reinforces the need for continuous investigations, considering different approaches and scales of analysis, in order to advance the understanding of the processes that govern landscape dynamics in the semi-arid region of Rio Grande do Norte.

## Supplementary Information

Below is the link to the electronic supplementary material.

ESM 1 Supplementary Material 1 (PDF 291 KB)

## Data Availability

All data supporting the findings of this study are available within the paper and its Supplementary Information.
