# Supplementary material for: Integrating morphometric and climatic variables into the mapping of land use and vegetation cover in the Caatinga biome
Source: Environ Monit Assess. 2026 Sep 29;198(10):1132. doi: 10.1007/s10661-026-15949-z (PMC13624018; doi:10.1007/s10661-026-15949-z)
Supplement: Supplementary file 1 [file 10661_2026_15949_MOESM1_ESM.pdf]

Supplementary Information

Table S1: Key to interpreting the mapping classes

Table S2: Predictor variables used in the model

Table S1 Key to interpreting the mapping classes

Level 2	Pattern Example (RGB654)	Description	Characteristics	Number of samples per class
**1.1** Urban Areas	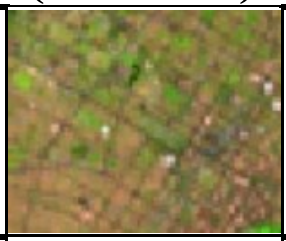	These are areas characterized by medium or high-density urban buildings.	It features light to medium gray tones, an irregular shape, a rough texture, and a checkered pattern.	1040
**1.2** Aquaculture	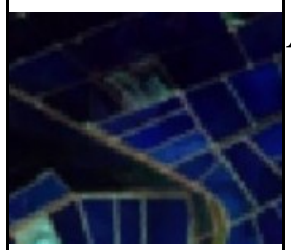	Area referring to artificial lakes intended primarily for the breeding of aquatic organisms for economic purposes. In addition to the practice of salt production.	It may feature shades of light blue or green and black. The texture is uniform and smooth, the pattern geometric and regular.	684
**2.1** Croplands	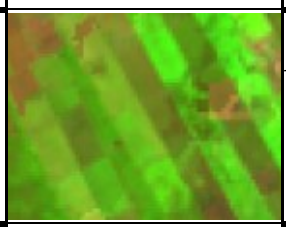	Areas identified as having temporary or permanent crops, irrigated or not.	It is characterized by its regular shape, light to medium green tones, and rough texture.	1195
**2.2** Pasture	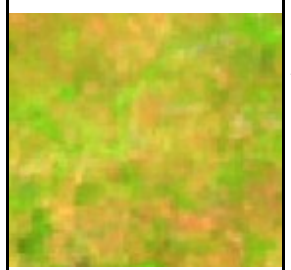	It includes native and planted pastures. These are environments under human interference, which may be for the grazing of cattle and other animals. Herbaceous vegetation can be found, including Grassland-Woody Savanna, Park Savanna-Steppe, interspersed with shrub species, as well as grassland formations.	It is characterized by its irregular shape and undefined pattern. It presents shades of light brown or light pink; however, depending on the humidity and health of the vegetation, it can display various shades of green.	1948
**3.1** Forest Vegetation	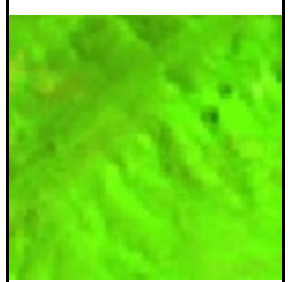	Savanna-steppe Forest areas, Semi-deciduous and Deciduous Seasonal Forest, with a predominance of continuous canopy, including riparian forest. Additionally, contact environments are represented by mixtures or interpenetrations of Savanna-Steppe and Seasonal Forest species.	It has an irregular shape, medium to dark green tones, an intermediate rough texture, and a non-systematic pattern.	556
**3.2** Savanna vegetation	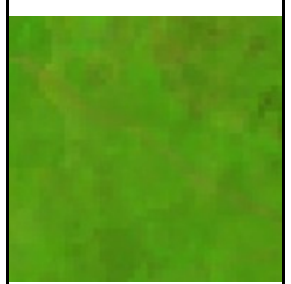	Vegetation with a predominantly shrubby stratum, sparsely distributed over a grassy-woody stratum. Similar to this category are Savanna-steppes, Wooded Savanna-steppes, and Shrubby Savanna-steppes. The savanna enclave (cerrado) was also included.	It is characterized by its irregular pattern, coarse texture, and light green tones.	1989
**3.3** Mangrove	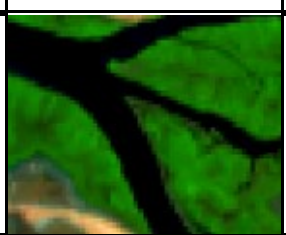	This refers to the coastal mangrove ecosystem, where the vegetation is dense and frequently flooded by the tide.	It features shades of dark green or olive green, with an irregular and rough texture.	179
3.4 Herbaceous Restinga (Sandbank Vegetation)	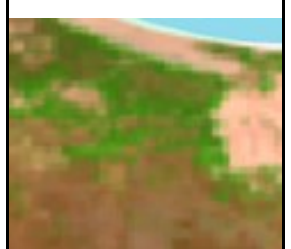	Herbaceous vegetation found in coastal sandy-water fields.	It has a green hue with exposed soil, a slightly rough texture, and an irregular pattern.	106
3.5 Apicum (Hypersaline Tidal Flat)	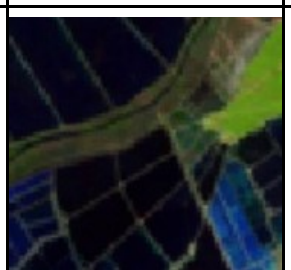	It is formed in saline and hypersaline areas, subject to sporadic flooding, in the transition to land or dune fields. It features vegetation known as halophytes.	It is characterized by shades of gray and light brown. It has an irregular pattern and a rough texture.	165
4.1 Rivers, Lakes, and Ocean	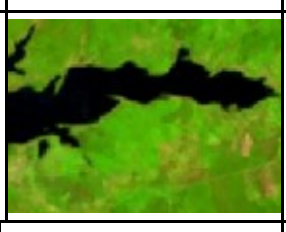	It refers to rivers, reservoirs, lakes, lagoons, and saltwater.	It is featured in shades of black or blue. Its pattern is well-defined with smooth texture.	723
5.1 Non-vegetated areas	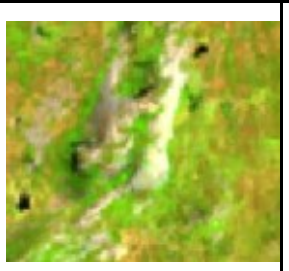	Areas with no vegetation cover, such as rocky outcrops, beaches, and dunes, both coastal and inland.	It is characterized by various color tones depending on the area, such as reddish, light brown, gray, and beige. The texture varies between	1223

			smooth and rough. Its pattern is undefined and scattered.	
5.2<br>Other uses	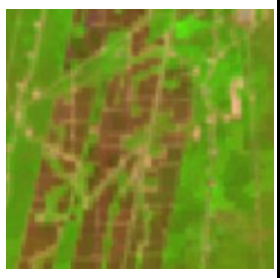	These correspond to industrial, commercial, communication network, mineral extraction, oil, and renewable energy (wind and solar farms) areas. Additionally, they include locations where it was not possible to identify land uses or where there are multiple uses, such as agriculture and pasture.	It features shades of gray, dark brown, or even black, with a defined geometric pattern.	1271
6		Areas blocked by clouds or atmospheric noise.	-	-

Table S2. Predictor variables used in the model.

Name (Variable)	Description	Formula	Reference
Normalized difference vegetation index (NDVI)	Estimation of the quantity and vigor of vegetation.	(NIR − RED) /(NIR + RED)	Rouse et al., 1974
Normalized difference water index (NDWI)	Water content estimate	(GREEN -NIR) / (GREEN + NIR)	Gao, 1996
Soil-adjusted vegetation index (SAVI)	Vegetation index adjusted to minimize the influence of soil in areas with little vegetation cover.	(NIR – RED) / (NIR + Red + L) × (1 + L); L = 0.5	Huete, 1988
Modified soil-adjusted vegetation index (mSAVI)	Modified version of SAVI to increase accuracy in exposed soils.	$[(2 * NIR + 1) - \sqrt{(2 * NIR + 1)} 2 - 8 * (NIR - RED)]/2$	Qi et al., 1994
Green soil-adjusted vegetation index (gSAVI)	SAVI version that uses the green band to highlight vegetation.	(NIR - GREEN) / ((NIR + GREEN+0.5)*(1+0.5))	Sripada et al., 2006
Normalized Difference Built-up Index (NDBI)	Index for identifying urban and built-up areas.	(SWIR - NIR) / (SWIR + NIR)	Zha et al., 2003
Green Normalized Difference Vegetation Index	NDVI version that uses the green band to assess vegetation.	(NIR - GREEN) / (NIR + GREEN)	Gitelson et al., 1996
Enhanced vegetation index (EVI)	Estimation of densely vegetated areas	2,5*(NIR-RED)/(NIR+6*RED-7,5*BLUE+1)	Liu & Huete, 1995
Difference vegetation index (DVI)	Simple vegetation index	NIR – Red	Tucker, 1979
Modified normalized difference water index (mNDWI)	A variant of NDWI, adjusted to identify water bodies.	(GREEN - SWIR) / (GREEN + SWIR)	Xu, 2006
Moisture stress index (MSI)	It measures water stress in vegetation.	SWIR1/NIR	Hunt Jr & Rock, 1989
Chlorophyll Vegetation Index (CVI)	Evaluates chlorophyll concentration.	(NIR / GREEN) * (RED / GREEN)	Vincini et al., 2008
Green Ratio Vegetation Index (GRVI)	Index based on the proportion of green to assess vegetation.	(NIR/GREEN)	Motohka et al., 2010
Modified Simple Ratio (MSR)	A modified version of the Simple Ratio for assessing vegetation health.	((NIR/RED)-1) / (sqrt(NIR/RED) + 1)	Chen, 1996
Ratio Vegetation Index (RVI)	Estimation of the proportion of vegetation	(NIR / RED)	Richardson & Wiegand, 1977
Normalized Burn Ratio (NBR)	Index used to detect burned areas.	(NIR-SWIR) / (NIR+SWIR)	Key & Benson, 2006
Normalized Difference Tillage Index (NDTI)	Index for detecting soil management practices, such as cultivation.	(SWIR1-SWIR2)/(SWIR1+SWIR2)	Van Deventer et al., 1997
Modified Photochemical Reflectance Index (MPRI)	Vegetation estimate.	(GREEN-RED)/(GREEN+RED)	Yang et al., 2008
MDE (Slope, hillshade, elevation, aspect)		-	SRTM
Spectral Linear Mixture Model (Fraction_solo, fraction_agua, fraction_vege)	Soil, water, and vegetation ratio.	-	Shimabukuro & Smith, 1991
Tasselled cap (vegetation, wetness, brightness)		-	Kauth & Thomas, 1976
Spectral Bands (B2, B3, B4, B5, B6)	Spectral bands of different wavelengths.	-	Landsat-8
Precipitation (precipitacao_2023)	Estimated precipitation data for 2023.	-	Silva et al., 2024
Land Surface Temperature (LST_2023)	Earth's surface temperature in 2023.	-	Jesus & Santana, 2017

Geographical Position (Latitude, longitude)	Measurements of position on the Earth's surface.	-	-
